# Triazine-based corrosion inhibitors: synthesis, significance, and emerging perspectives

**DOI:** 10.1039/d5ra09707j

**Published:** 2026-06-04

**Authors:** Ihab Shawish, Hessa H. Al-Rasheed, Assem Barakat, Hassan H. Hammud, Ayman El-Faham

**Affiliations:** a Department of Math and Sciences, College of Sciences and Humanities, Prince Sultan University Riyadh 11586 Saudi Arabia ishawish@psu.edu.sa; b Department of Chemistry, College of Science, King Saud University P.O. Box 2455 Riyadh 11451 Saudi Arabia halbahli@ksu.edu.sa ambarakat@ksu.edu.sa; c Department of Chemistry, College of Science, King Faisal University Al-Ahsa 31982 Saudi Arabia hhammoud@kfu.edu.sa; d Department of Basic Medical Sciences, College of Medicine, Dar Al Uloom University Riyadh 11512 Saudi Arabia ayman.a@dau.edu.sa; e Department of Chemistry, Faculty of Science, Alexandria University P.O. Box 426 Ibrahimia Alexandria 21321 Egypt ayman.elfaham@alexu.edu.eg

## Abstract

Triazine is a nitrogen-containing heterocyclic moiety that represents a significant building block, which is prevalent in a diverse array of pharmaceutical agents and natural substances. Numerous triazine derivatives have been identified as effective corrosion inhibitors, attributed to the configuration of three nitrogen atoms that are symmetrically positioned within a six-membered ring structure. Moreover, the lone pair electrons on the nitrogen atoms, along with the influence of ring substituents, markedly enhance the adsorption of triazine-based compounds onto metallic surfaces, thereby contributing to their efficiency as corrosion inhibitors. This review offers a comprehensive outline on the synthetic methodologies and various applications related to triazines, accompanied by a critical literature review focusing on the corrosion inhibition efficacy of triazine derivatives. Additionally, investigations insights regarding the adsorption of triazines onto metallic surfaces, derived from both experimental and computational investigations, are delineated in this article. Furthermore, the challenges and future perspectives are explored to provide valuable benefit for researchers in this field.

## Abstract 2

Triazine based compounds are promising corrosion inhibitors due to their nitrogen rich structure, which promotes strong adsorption on metal surface.

## Graphical abstract

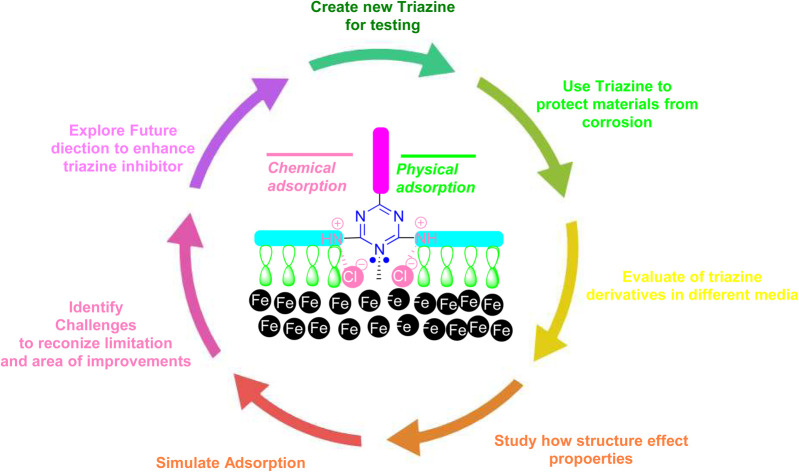

## 1 Introduction

**Fig. 1:**
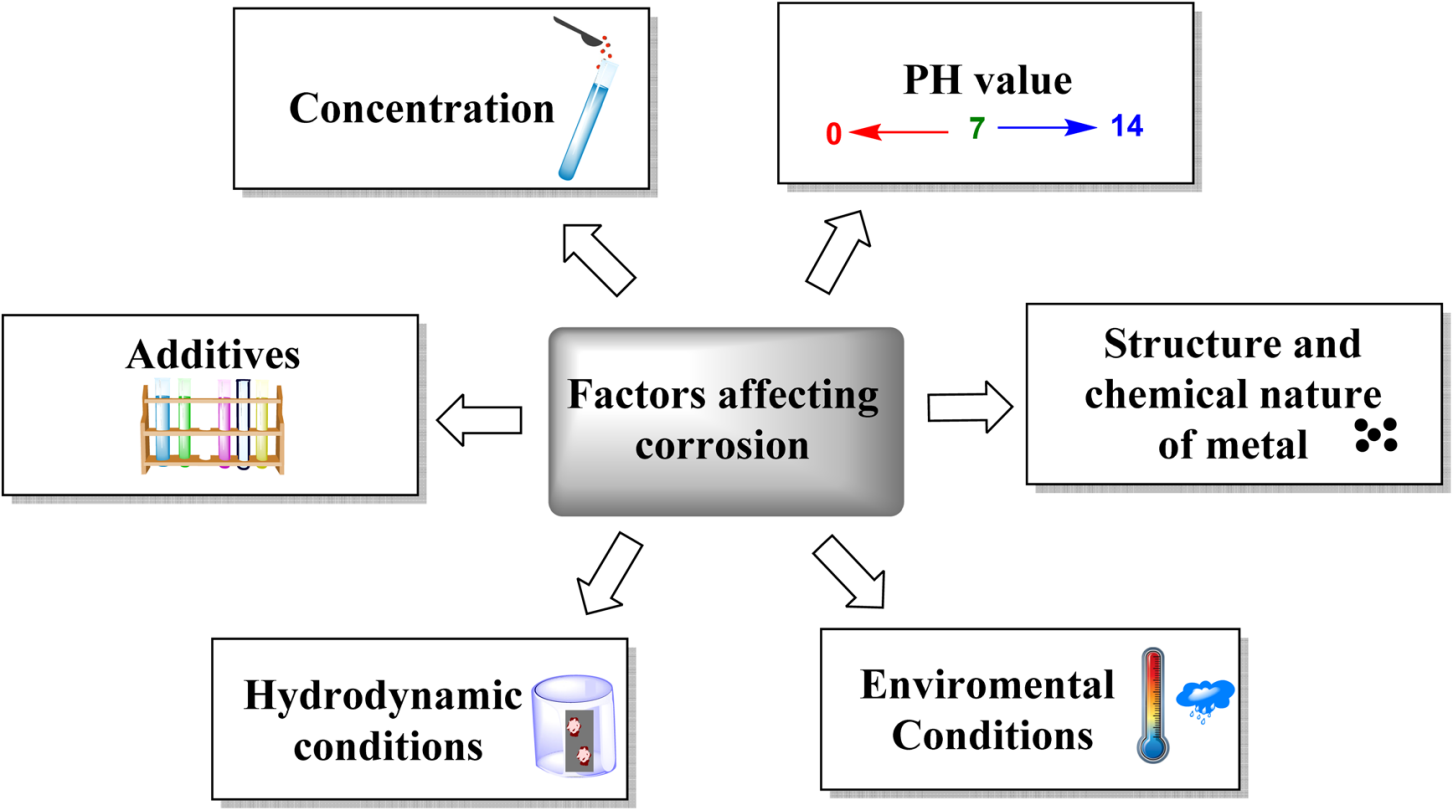
Some factors affecting the corrosion of metallic materials.

Corrosion represents a significantly deleterious and intricate phenomenon through which materials, especially metals and their respective alloys, experience structural deterioration because of interaction with several environmental factors, as shown in Fig. 1.^1,2^ Over the past decades, several studies worldwide have explored the cost of corrosion and its impact on national economies. For example, a study by the National Association of Corrosion Engineers (NACE) in 2002 estimated that the United States loses about $276 billion annually due to corrosion, equating to approximately 3.1% of the nation's gross domestic product (GDP).^3,4^ By 2011, the cost of corrosion in the U.S. had surpassed $2.2 trillion. In India, the 1st Global Corrosion Summit held in New Delhi reported that corrosion caused losses of about $45 billion in year 2011.^3,4^

As per the latest assessment conducted by NACE, the economic cost of corrosion (COC) currently exceeds US $2.5 trillion, constituting approximately 3.4% of the global economic landscape.^5,6^ The COC can be systematically categorized into five major sectors: production and manufacturing, governmental entities, utilities, transportation, and infrastructure.^6,7^ Numerous sectors, particularly the oil, gas, and petroleum industries, are profoundly impacted by corrosion-related failures. Corrosion phenomena occurring during refining and transportation processes generate numerous challenges for the oil and gas sectors. The economic consequences of corrosion extend to design expenditures, operational costs, capital investments, and control measures. Moreover, various industrial methodologies, including acidization, surface descaling, acid pickling, oil-well acidification, and electrolytic cleaning, involve the use of highly aggressive cleaning agents referred to as electrolytes, which are associated with significant safety and financial implications.^8,9^ Beyond the apparent effects, corrosion leads to plant shutdowns, resource depletion, product loss or contamination, decreased efficiency, and excessive maintenance and overdesign costs. It also poses serious safety risks and can hinder technological progress. Given the significant economic, safety, and environmental challenges associated with corrosion and its potential consequences, numerous preventive practices have historically been developed and implemented, while ongoing investigations continue to seek improved solutions.^4,10^

Acidic solutions are broadly used to eliminate rust and scales from corroded mild metal for further applications such as rolling and galvanizing. Aqueous acidic solutions are employed for de-scaling, acid pickling, and acid treatment. However, the exposure of metal to these solutions can induce corrosion, creating substantial safety and economic risks.^11,12^

To mitigate this, corrosion inhibitors are utilized to reduce the rate of corrosion and protect metal from further degradation. Workers in related industries must identify effective corrosion inhibitors for protection of metals from aggressive dissolution. Corrosion inhibitors typically contain electron-rich elements such as nitrogen, oxygen, sulfur, phosphorus, or multiple bonds and aromatic rings, which provide physical or chemical adsorption sites on the metal surface.^13–16^ Moreover, from an environmental perspective, it is preferable for corrosion inhibitors to be non-toxic and biodegradable.^17,18^

Several strategies are currently employed to mitigate corrosion-related deterioration. This include the application of protective anti-corrosion coatings, the development and utilization of corrosion-resistant alloys, and the employment of corrosion inhibitors. A corrosion inhibitor is an additive that, at low concentration, substantially slows the rate of corrosion in a given corrosive environment without changing the composition of the corrosive medium. These inhibitors are widely used across various industrial sectors, such as boilers systems, the sugar industry, oil and gas, the petroleum, water treatment facilities, chemical reactors, and the energy industry.^19^

**Fig. 2:**
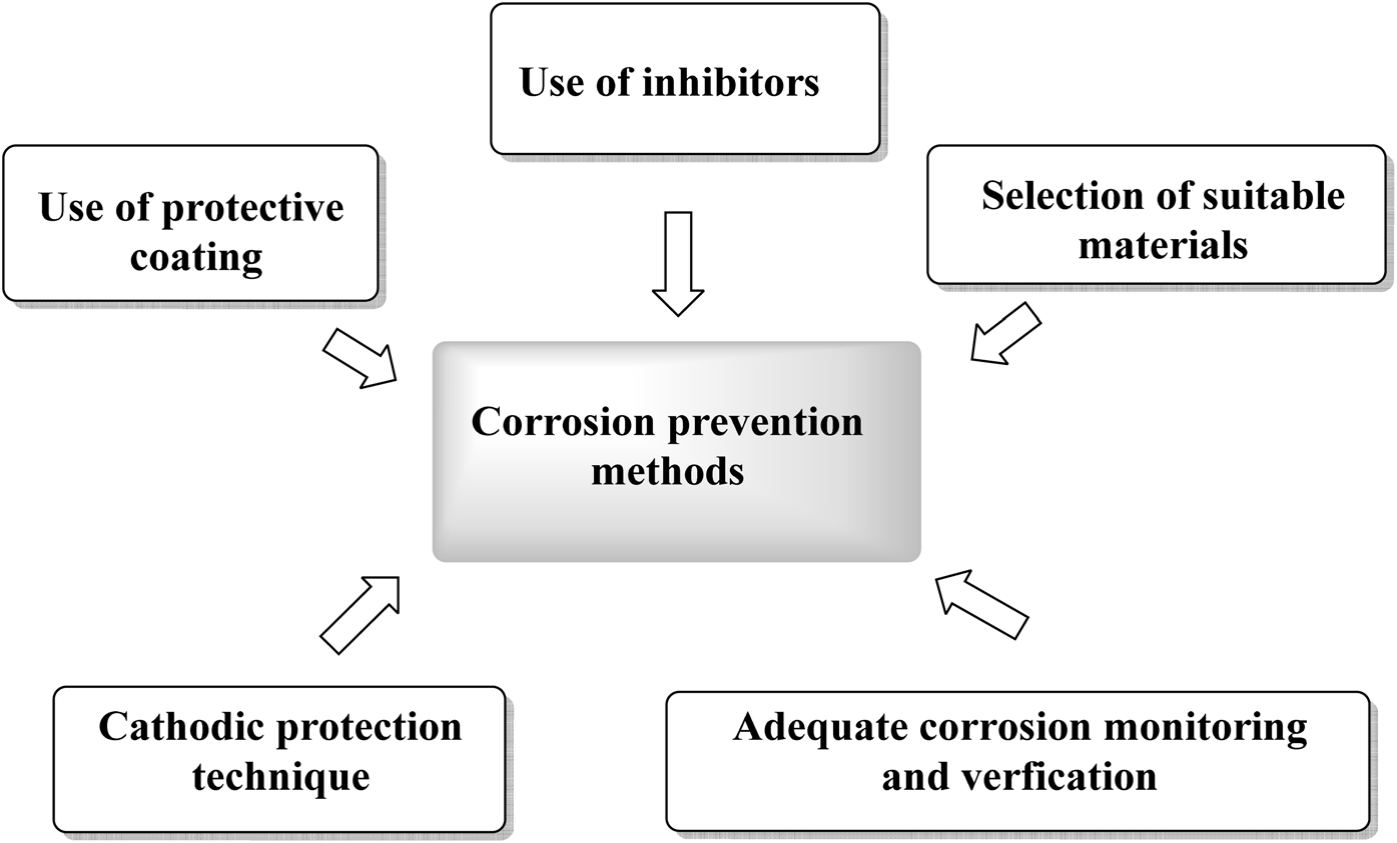
Some corrosion prevention methods.

Corrosion prevention methods comprise systematic protocols and approaches aimed to reduce the losses and damage caused by corrosion on the industrial scale (Fig. 2).^20,21^

The effectiveness of each method depends on many factors such as corrosion type, metal substrate, pH of the medium, temperature and other related aspects. Consequently, a variety of protocols have been developed to mitigate corrosion, including design modification,^22^ sacrificial anodes^23,24^ and surface coating.^25–27^ Surface coating approaches function by the forming of a protective layer to the metal and environmental conditions; this protection is achieved through adsorption of the coating material onto the metal surface. Several reported coating and inhibitor approaches include nanocomposites,^28^ inorganic compounds,^29,30^ and plant-derived extracts^31^ which offer environmentally friendly and cost-effective protection with broad applicability and long-lasting performance. However, several studies have raised toxicity concerns for certain inhibitors at high concentrations.^32–34^

Among N-heterocyclic molecules, numerous molecules such as imidazole, pyridine, quinolone, purine, and pyrimidine and their derivatives have demonstrated remarkable anticorrosive performance.^16,35,36^ These heterocyclic systems are widely regarded as effective organic corrosion inhibitors because of their structural features, including multiple heteroatoms, conjugated π-bonds, and aromatic rings. In addition, the presence of polar functional groups and bulky molecular frameworks or long alkyl chains are expected to enhance their adsorption efficiency on the metallic substrate.^37–39^ Furthermore, researchers have investigated structurally diverse heterocyclic organic molecules as effective agents for corrosion inhibition. These compounds possess a π-bond bonding system and include heteroatoms such as N, O, and S. The unique properties of these compounds enhance their ability to adsorb onto metal surface, which significantly boosts their corrosion efficacy. As a result, they play a vital role in protecting metals from corrosion.^16,36,40–48^

In this context, the six-membered aromatic nitrogen heterocycle of triazine serves as a key scaffold for corrosion-inhibiting compounds. Extensive investigation into this class has highlighted derivatives of 1,3,5-triazine (*s*-triazine) as the most prominent. Indeed, substituted triazines at both nitrogen and carbon positions are extensively utilized in industrial applications. These organic molecules protect metals by adsorbing them onto their surfaces and forming thin protective films.

This article provides an overview of triazine-based heterocyclic systems, their role in corrosion prevention, challenges, and future perspectives.

### 1.1. Triazines, synthesis and their applications on corrosion protection

**Fig. 3:**
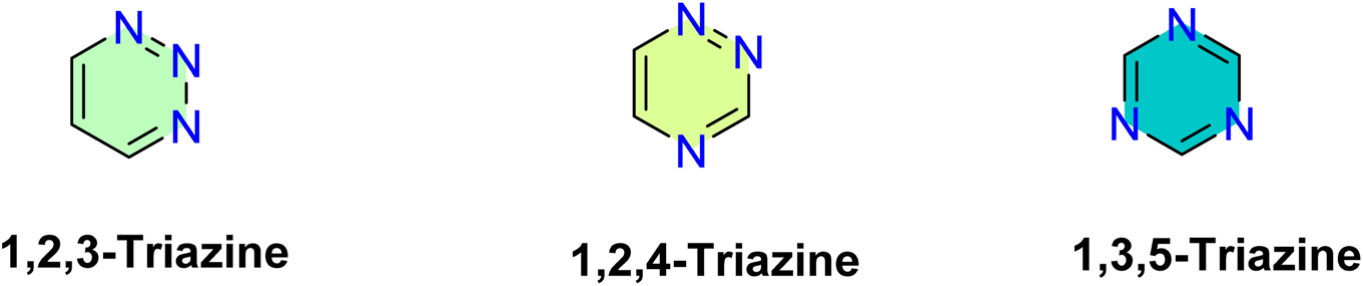
Structure of isomers of triazine.

Both triazine ring (C_3_H_3_N_3_) and benzene ring (C_6_H_6_) exhibit structural similarity, with three carbon atoms replaced by nitrogen atoms.^49^ Depending on the spatial arrangement of the nitrogen atoms, three isomers of triazine can be identified: 1,2,3-triazine, 1,2,4-triazine, and 1,3,5-triazine, as illustrated in Fig. 3.^50^ Among the various triazine isomers, 1,3,5-triazine (symmetric, *s*-triazine) is the most common and widely referred investigated structural, while the others are classified as asymmetric triazines (Fig. 3).

1,3,5-Triazines serve as the backbone of many agrochemicals, such as herbicides, fungicides, and insecticides.^51^ Beyond agriculture, these derivatives find applications in analytical chemistry (as complexation agents),^52^ electrochemistry (as redox-active systems),^53^ and as templates for covalent organic framework.^54,55^

Recently, efforts have focused on the application of cyanuric chloride for the synthesis of *s*-triazine derivatives due to its utility in environmentally friendly organic synthesis under mild conditions with low-cost reagents. These derivatives have also shown promising pharmacological potential, with reported antimicrobial, antiviral, antimalarial, anticancer, anti-inflammatory, and antitubercular activities, as well as roles in treating diseases like Alzheimer's and autoimmune disorders.^56^ Additionally, they have been explored for anti-methamphetamine, anti-HIV, anti-angiogenic, and anti-trypanosomal applications. They are further applied as scaffolds in medicinal chemistry.^56^

#### 1.1.1. Synthesis of *s*-triazine derivatives

**Scheme 1:**
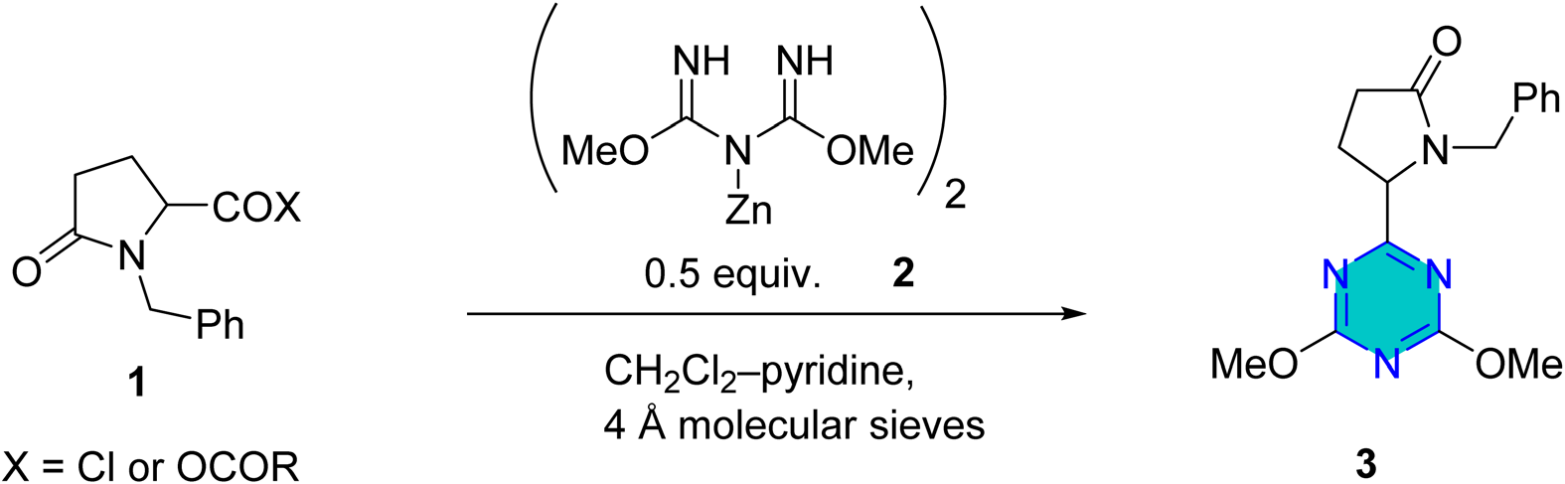
6-Substituted 2,4-dimethoxy-1,3,5-triazines.

Several reported protocols have been employed for preparation of *s*-triazine derivatives from inexpensive and readily accessible starting materials.^56,57^ For example, Rembarz *et al.*^58^ reported syntheses of 6- substituted 2,4-dimethoxy-1,3,5-triazines (3) from the reaction of activated carboxy groups (1, acid chlorides, anhydrides, acylimidazolides) with zinc dimethyl imidodicarbonimidate salt (2) (Scheme 1). In this method, the rate of conversion of the activated carboxy group was low, while good yields obtained when used a very large excess of the carboxylic acid derivative.^58^

Later, Oudir *et al.*^59^ repeated the same reaction under same conditions, the authors reported a moderate yield (53%) of 6-substituted 2,4-dimethoxy triazine derivatives (3) when stoichiometric amount of the acid chloride was used. In addition, they claimed that the moderate yield obtained because of the hydrolysis of the acid chloride by water formed during the reaction. They run the reaction again using different solvents in the presence of 4 Å molecular sieves to avoid hydrolysis. Higher yields were obtained when acid chloride reacted with the salt (2) in the presence of 4 Å molecular sieves and using mixed solvent DCM-pyridine as shown in Scheme 1.^59^

**Scheme 2:**
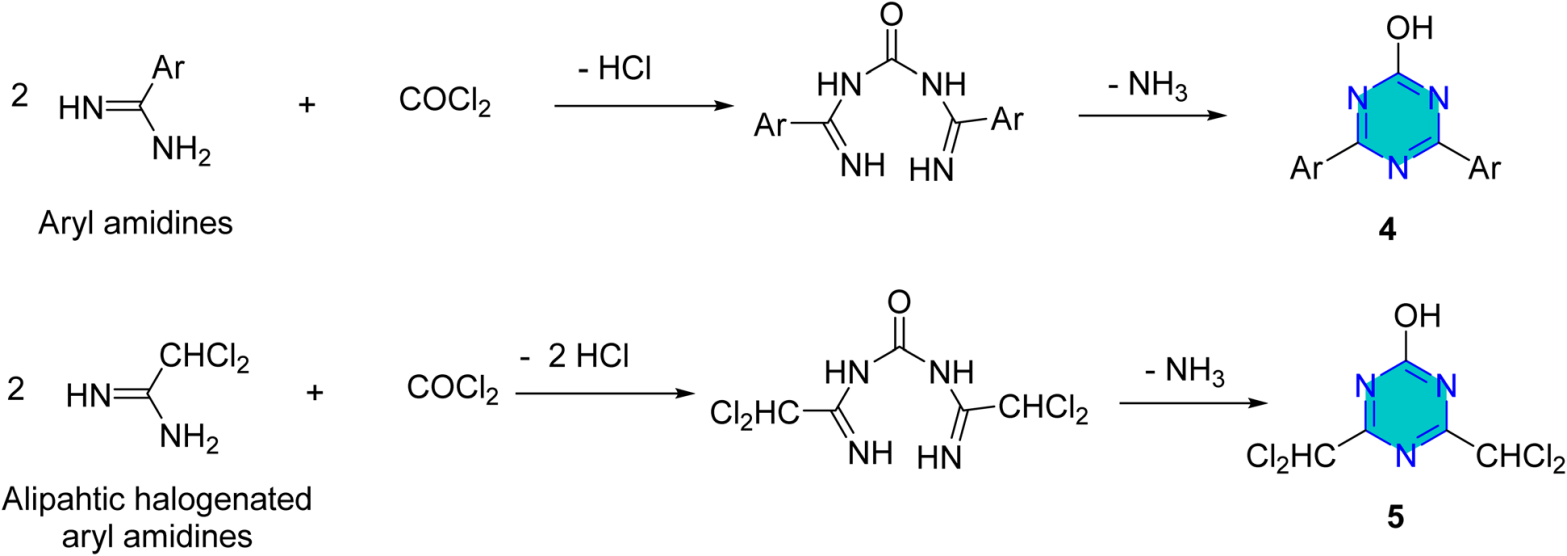
Synthesis of 2-hydroxy-4,6-diaryl-*s*-triazine derivatives.

Kumar *et al.*^60^ reported the preparation of 2-hydroxy-4,6-diaryl-*s*-triazine derivatives (4) and 2-hydroxy-4,6-dichloromethyl-*s*-triazine derivatives (5) from the reaction of the aryl amidines and halogenated aliphatic amidines with phosgene, respectively to afford the desired products 4 and 5 (Scheme 2).^60^

**Scheme 3:**
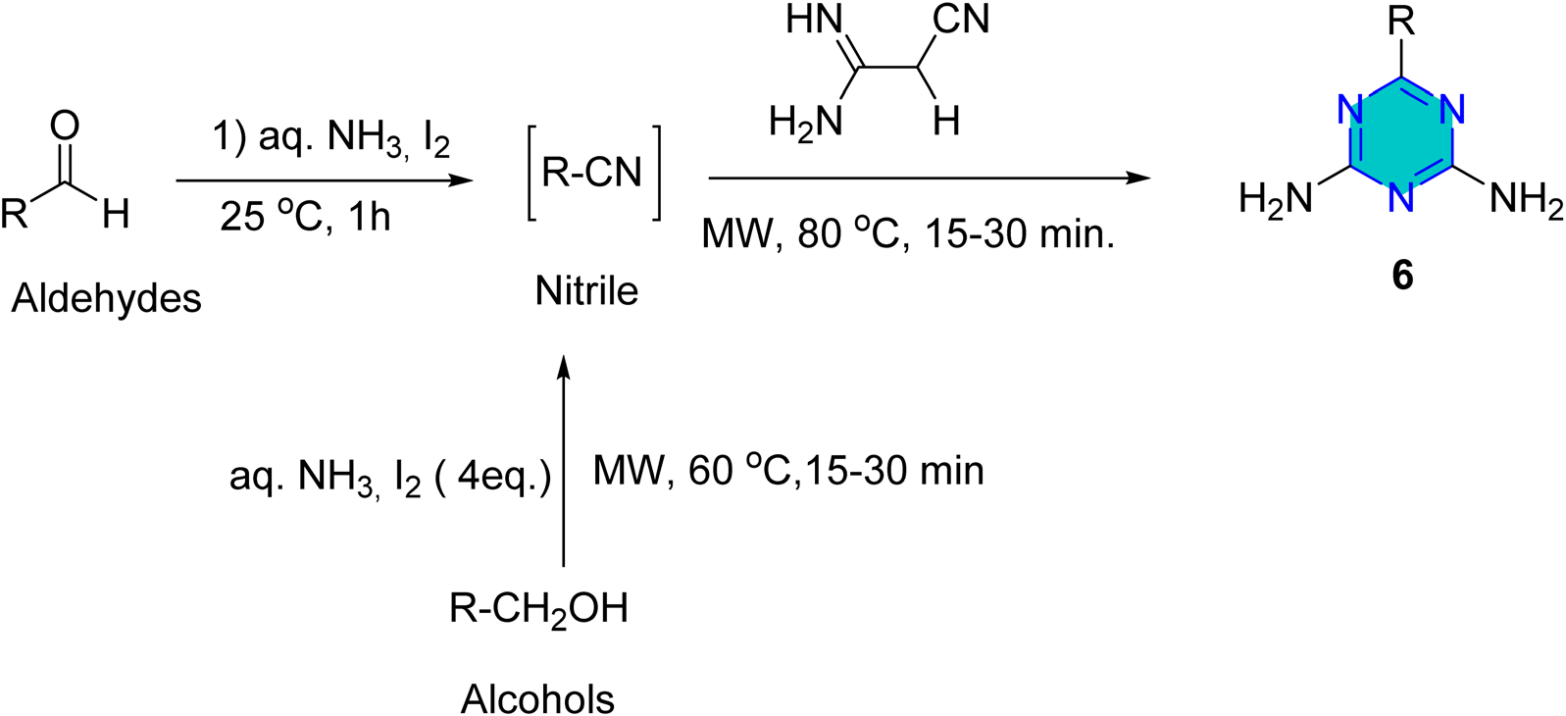
Microwave synthesis of *s*-triazine derivatives.

The synthesis of *s*-triazine derivatives (6) employing microwave irradiation was reported by Shie *et al.*^61^ In this reaction primary alcohols or aldehydes was reacted with iodine in ammonia–water to generate nitriles intermediate, which without isolation reacted with dicyandiamide to give the corresponding *s*-diamino-*s*-triazines derivatives (6) in high yield (Scheme 3).^61^

**Scheme 4:**
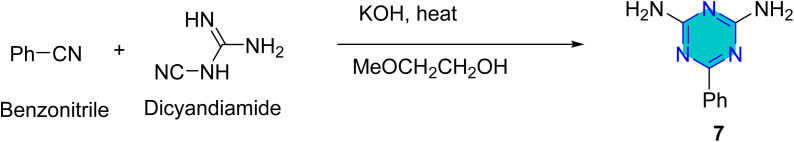
Synthesis of 3,5-diamino-*s*-triazine derivatives.

Simons and Saxton reported the synthesis of diamino-*s*-triazine derivatives (7) from the reaction of dicyandiamide with benzonitrile in the presence of KOH as shown in Scheme 4.^62^

**Scheme 5:**
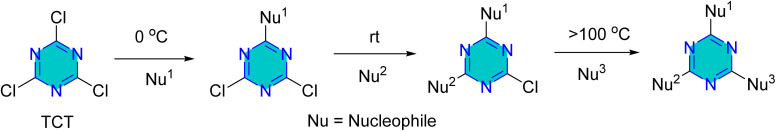
Nucleophilic substitution reaction of TCT.

Other derivatives of *s*-triazine have also been reported using commercially available materials.^61,62^ In addition to the direct availability of triazine derivatives, these compounds can now be easily synthesized from the readily accessible, inexpensive precursor cyanuric chloride (1,3,5-trichlorotriazine; TCT) (Scheme 5). TCT, having three reactive chlorines, serves as a useful and versatile precursor for synthesizing numerous lead compounds, various multitopic molecules, and molecular hybrids featuring *s*-triazine as a core scaffold. Any nucleophilic species (N, O, or S) can be used to replace chlorine atoms from TCT *via* S_N_Ar mechanism under different base and temperature conditions.^57,63^

This protocol has been used for the synthesis of the trisubstituted 1,3,5-triazines, even the uncondensed derivatives bearing one or more amino groups at 2, 4 or 6 positions have been extensively described for their potential applications.^63^

#### 1.1.2. Triazine in corrosion inhibition

##### 1.1.2.1. Limitations in traditional organic corrosion inhibitors

The organic corrosion inhibitors that are commonly utilized within various industrial sectors include imidazolines, acetylenic alcohols, amides, amines, quaternary ammonium salts, among others. The chemistry of their synthesis involves a lengthy process characterized by complex synthetic steps, which not only require considerable time but also suffer significant financial costs.^16^

Furthermore, the isolation and purification of certain inhibitors present substantial challenges. In addition, the release of these inhibitors into planetary and aquatic ecosystems poses a significant environmental risk. Considering these concerns, several guidelines have been established by international regulatory frames regarding the implementation of environmentally benign practices and reagents for the formulation of organic corrosion inhibitors.^64,65^ Presently, research efforts related to the preparation of organic corrosion inhibitors are increasingly directed towards adopting Green Chemistry Principles^66^ and applying Green Chemistry Metrics^67^ in response to environmental considerations. These methodologies supported the usage of environmentally benign solvents, solvent-free synthesis, the incorporation of biocatalysts, the use of single-step modern synthetic methodologies such as multicomponent reactions (MCRs),^68^ and application of ultrasonic and microwave irradiation techniques for the synthesis of inhibitors.^69,70^ Using MCRs offer economy reduced reactions steps, and structural diversity, also the ultrasonic, and microwave irradiation can significantly shorten reaction times, enhance the yield, and lower energy. This is on the laboratory scale and may be present challenges in terms of scale-up, reproducibility, and industrial scale. In addition, the translations of MCRs, as well as ultrasonic-microwave-assisted techniques from other area of chemistry into corrosion-inhibitor design is not always straightforward.^70^ Where corrosion inhibitors require specific functional motifs to insure strong and stable interactions with metal surfaces. These structural requirements may not always be readily accommodated within multicomponent platforms, which can limit their applicability and partly explain the relatively limited their use.

Furthermore, alternative corrosion inhibitors that are more environmentally sustainable have also been proposed, derived from categories such as natural extracts,^71^ amino acids,^72^ ionic liquids,^3^ pharmaceutically active compounds,^73^ macrocyclic compounds,^74^ biopolymers, and others.^75^

##### 1.1.2.2. Significance of triazines derivatives in corrosion inhibition

Organic corrosion inhibitors operate through the mechanism of adsorption onto a specified metallic surface, either through electrostatic attraction (physical adsorption), or through the sharing of lone pairs of electrons, which may occur through σ-bonding or π-back bonding, or *via* back bonding, such as hydrogen bonding (chemical adsorption).^76^ This process culminates in the establishment of a thin protective film, which serves as a barrier to resist the corrosion from the corrosive electrolyte in the medium. From a performance-oriented perspective, organic molecules containing nitrogen are traditionally regarded as efficacious inhibitors.^77,78^ Heterocyclic compounds belonging to the azole's family (such as, pyrazole, imidazole), pyridines, pyrimidines, and their benzene-fused derivatives are frequently utilized as corrosion inhibitors.^49^

Triazine molecule is characterized by the existence of three nitrogen atoms arranged in various configurations, as previously discussed (Fig. 3). Nevertheless, none of the native forms, such as 1,2,3-triazines, 1,2,4-triazines, or 1,3,5-triazines, have proven effective as corrosion inhibitors.^18^

The presence of substituents significantly influences their inhibitive performance. Electron-donating moieties (*e.g.*, alkoxy, hydroxyl, amino) can enhance metal-inhibitor bonding by increasing electron density at donor regions, whereas electron-withdrawing groups (*e.g.*, cyano, carboxyl, nitro) can reduce inhibition activity.^4^ These molecules possess a greater number of active sites for interaction with the metallic surface and exhibit an increased geometric surface area, which enhances surface coating. Amongst the heterocyclic organic molecules, *s*-triazines constitute a significant class, and a variety of triazine-based corrosion inhibitors have been investigated in both acidic and neutral corrosive environments. Consequently, numerous functionalized triazine derivatives have been synthesized and are employed as corrosion inhibitors.

**Fig. 4:**
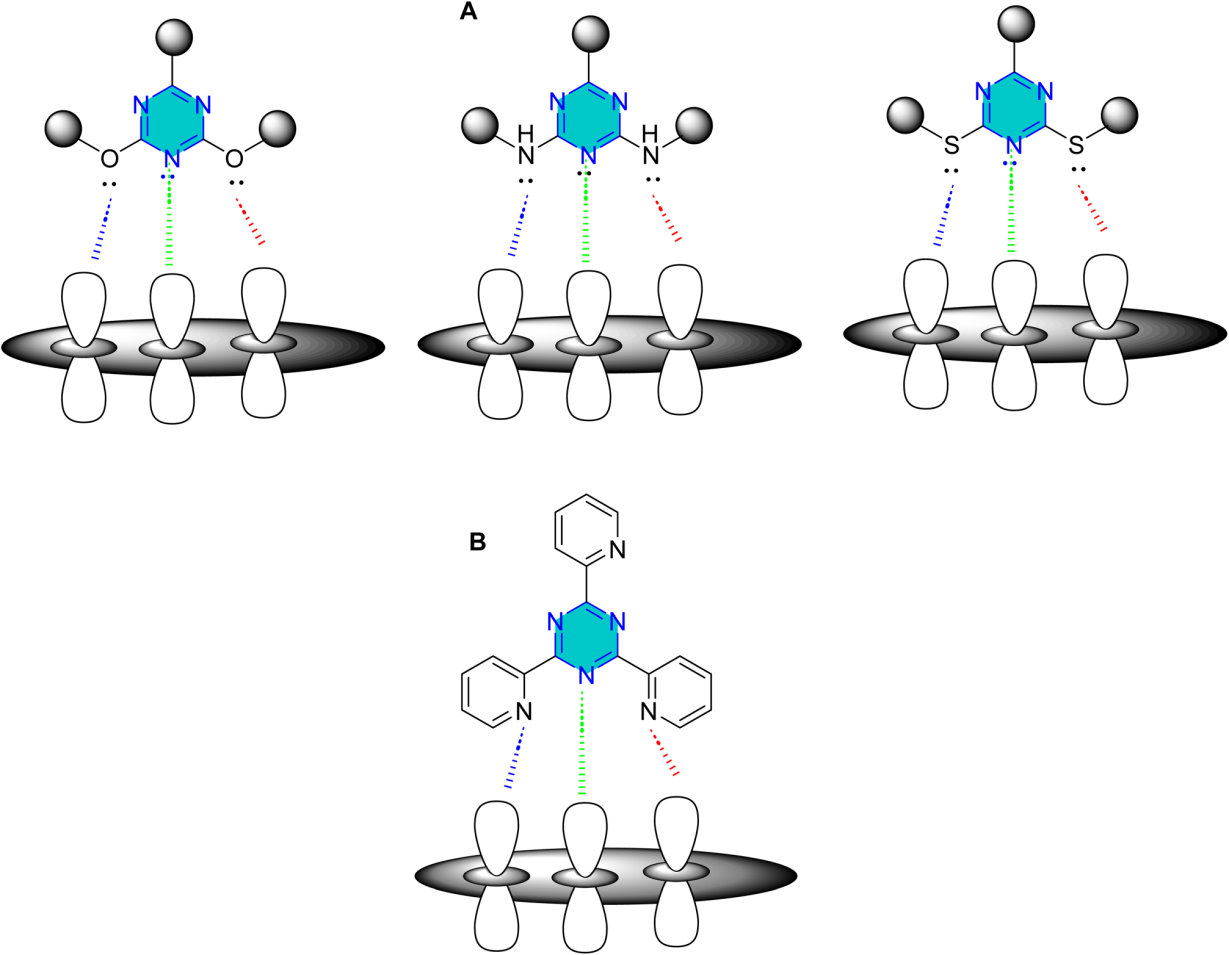
(A) The effect of substituents on adsorption properties of triazine derivatives side chain coordination. (B) Effect of substituents on minor, middle and major coordination; green = monodentate (minor coordination), green and blue = bidentate (middle coordination) and green, blue and red = polydentate ligand (major coordination).

*s*-Triazine derivatives can act as monodentate, bidentate, or polydentate ligands. For instance, an unsubstituted *s*-triazine can adsorb flatly as a tridentate ligand, but bulkier groups may reduce its coordination contribution, causing it to behave as bidentate or monodentate. Triazine derivatives with heteroatoms (N, O, or S) in their side chains can also function as polydentate ligands, forming chelating complexes (Fig. 4).^7^

#### 1.1.3. Triazine-based corrosion inhibitors in diverse corrosive medium

##### 1.1.3.1. Triazine-based corrosion inhibitors in acidic medium

The steel–acid interface has become a focal point for research aimed at assessing and investigating organic corrosion inhibitors. Numerous reports describe the effectiveness of triazines as corrosion inhibitors for steels when exposed to different acidic environments.^79^ Functionalized triazine derivatives, rather than their native forms (1,2,3-triazines, 1,2,4-triazines, or 1,3,5-triazines), are necessary for effective corrosion inhibition. This is accomplished by chemical functionalization of the triazine ring, specifically at the C or N atoms. Researcher modify the structure by adding alkyl chain lengths of varying lengths, introducing electron-donating or withdrawing substituent groups, and incorporation additional heteroatoms or phenyl rings. These modifications increase the number of active sites for interaction with the metal substrate and provide better geometrical surface area, leading to improved surface adsorption and coverage.^79^

**Fig. 5:**
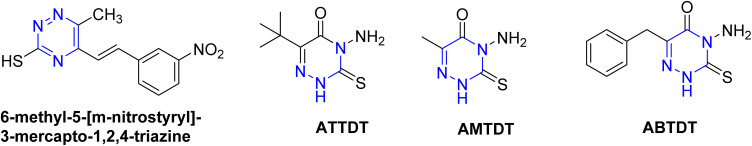
Structure of 1,2,4-triazine derivatives reported as corrosion inhibitors.

Migahed and Nassar reported the synthesis of 6-methyl-5-[*m*-nitrostyryl]-3-mercapto-1,2,4-triazine (Fig. 5) and evaluated their corrosion inhibition activity for mild steel in 12% HCl at 50 °C using many chemical and electrochemical techniques.^80^ The results indicated a significant reduction in the anodic dissolution of metal steel. This strong adsorption capacity of the synthesized compound can be linked to the presence of multiple nitrogen adsorption centers along with various donor moieties. Electrochemical impedance spectroscopy (EIS) results exhibited a decrease in the double layer capacitance (*C*_dl_) and an increase in charge transfer resistance (*R*_ct_) and inhibition efficiency (*η*%) with higher inhibitor concentrations, indicating a thicker electrical double layer.

John and Joseph examined the activity of three triazines, AMTDT, ATTDT, and ABTDT (Fig. 5) as corrosion inhibitors for mild steel in 1 M HCl.^81^ ABTDT, with a –CH_2_Ph substituent, showed high efficiency (99.90%) at a concentration of 200 mg L^−1^. All three-derivative exhibited cathodic predominance, and their adsorption behavior was found to conform to the Langmuir adsorption isotherm. The nitrogen atoms and π-donor moieties were identified as major adsorption centers.^81^

**Fig. 6:**
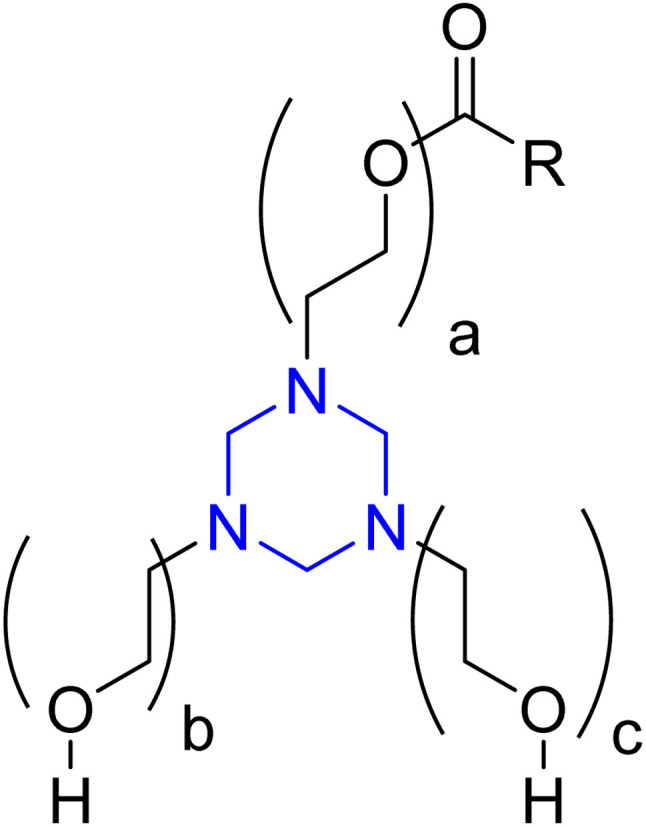
Structure of 1,3,5-triethanolhexahydro-1,3,5-triazine; where: *a* + *b* + *c* = 9 (I), 13 (II) and 23 (III); R from oleic acid.

Al-Sabagh *et al.* reported three nonionic surfactants containing a 1,3,5-triethanolhexahydro-1,3,5-triazine core (Fig. 6) and assessed them as inhibitors for mild steel corrosion in 1 M HCl, with theoretical studies highlighting the involvement of N and O atoms in their inhibition. The authors additionally evaluated the surface tension of the inhibitors at concentrations below and above the critical micelle concentration (CMC).^82^

**Fig. 7:**
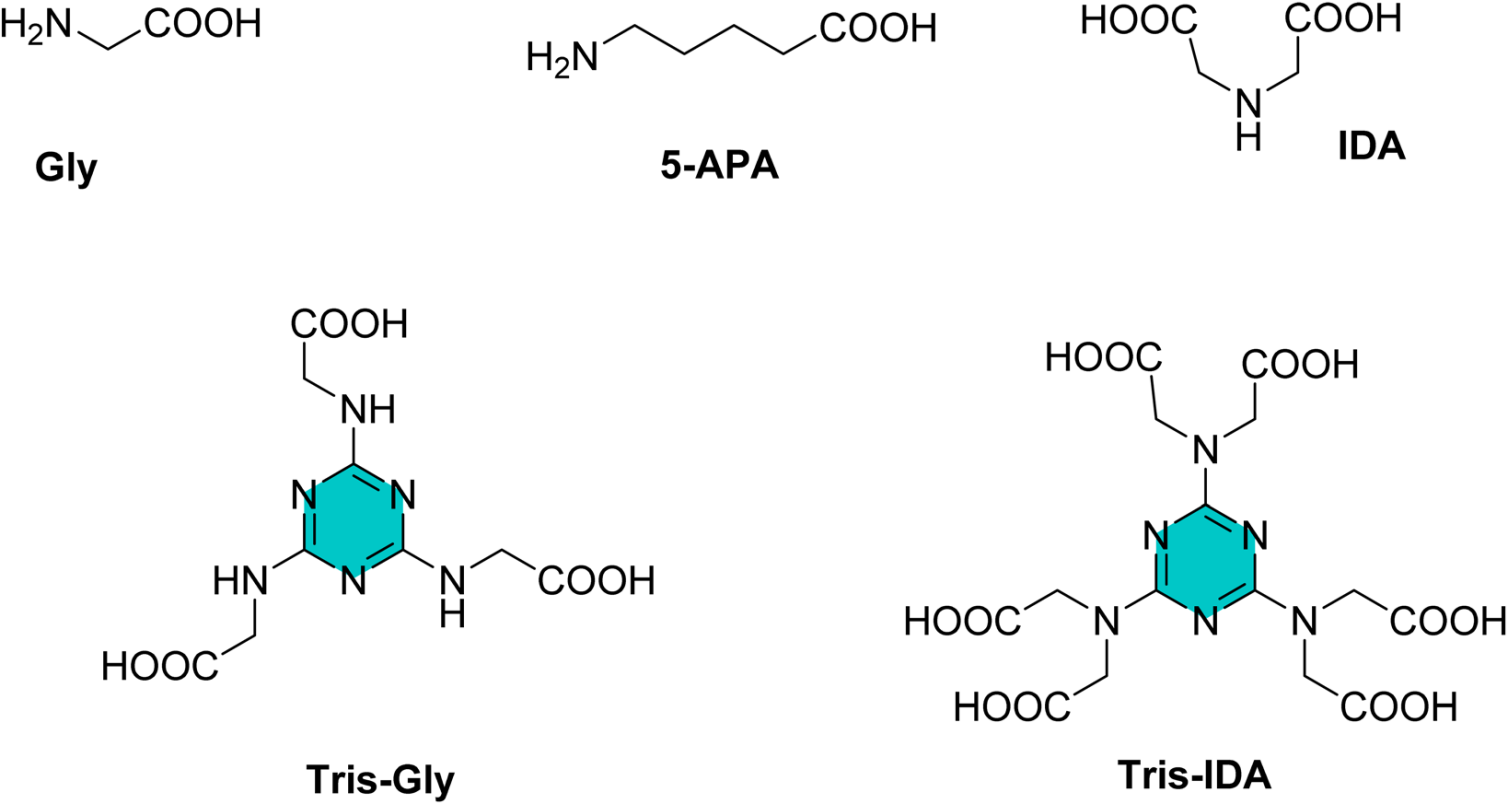
Structure of *s*-triazine amino acid derivatives.

Yoo *et al.* examined corrosion inhibition performance of three amino acids, glycine (Gly), 2,2′-azanediyldiacetic acid (IDA), 5-aminopentanoic acid (5-APA) in comparison with two triazine (Tris) derivatives (Fig. 7). Their finding demonstrated that the incorporation of the triazine ring markedly enhanced the inhibition efficiency.^83^ Overall, the results showed that the presence of the *s*-triazine moiety within the inhibitor structure plays a pivotal role in enhancing corrosion protection.

**Fig. 8:**
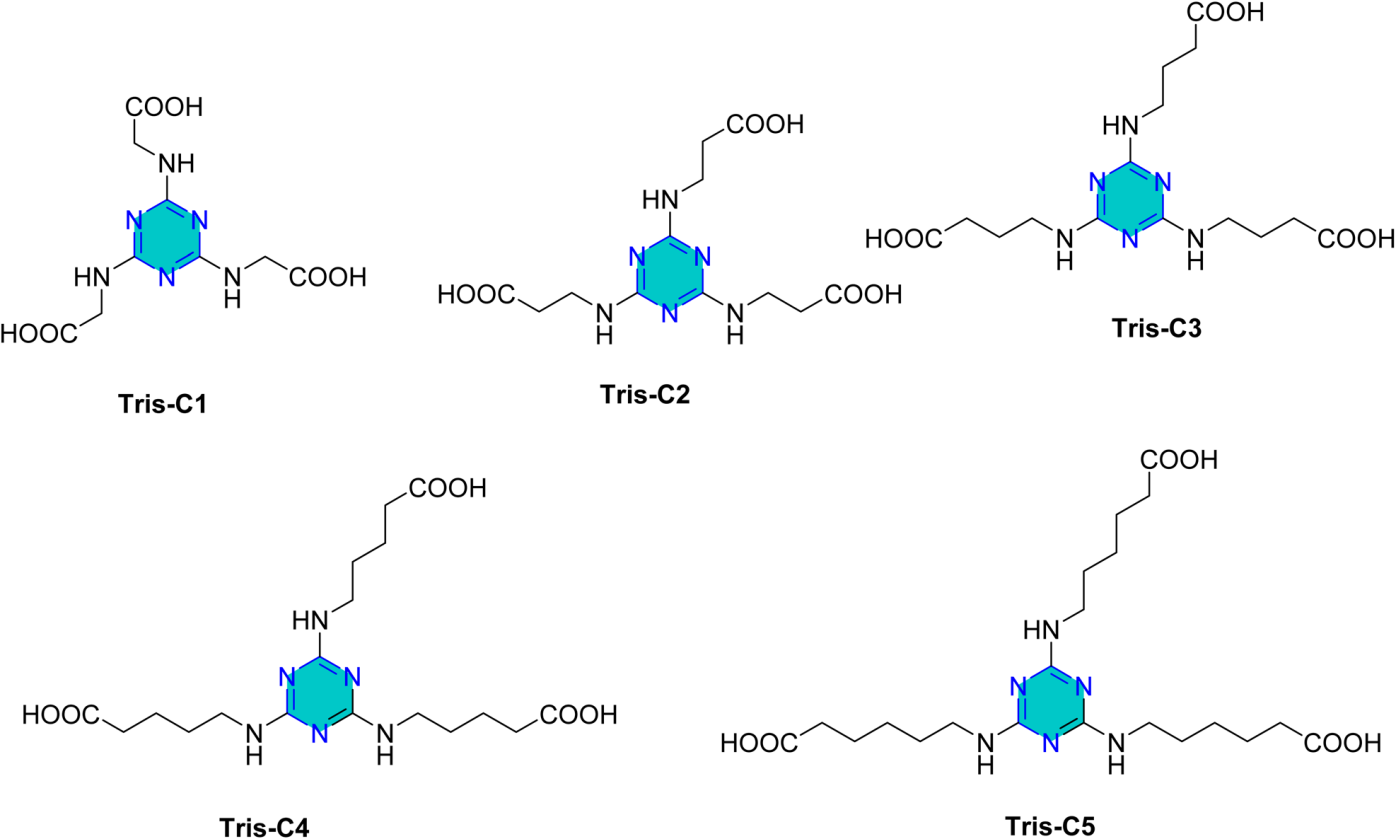
Structures of 2,4,6-tris(*n*-carboxyalkylamino)-1,3,5-triazine derivatives as corrosion inhibitors for mild steel in acid medium.

The same research group subsequently evaluated a series of five 2,4,6-tris(*n*-carboxyalkylamino)-1,3,5-triazine derivatives (Tris-C_*n*_, Fig. 8) as corrosion inhibitors for mild steel in 1 M HCl using electrochemical techniques. The authors suggest that Tris-C_*n*_ derivatives act as mixed type inhibitors that competitively reduce both anodic and cathodic reactions. They observed that as alkyl chain length and concentration of Tris-C_*n*_ increased, the corrosion current density decreased, while the (*η*%) increased. This trend was also similar in electrochemical impedance spectroscopy (EIS) data, which showed increasing in charge transfer resistance (*R*_ct_) alongside decreasing in the double layer capacitance (*C*_dl_). Based on these results, the authors claimed that Tris-C_*n*_ with longer alkyl chains enhance the protective layer on the metal surface against corrosion. This improvement is attributed to the thicker and denser protective layer resulting from the longer alkyl chains. Additionally, they proposed that the adsorption of Tris-C_*n*_ derivatives follows the Langmuir isotherm adsorption model. Finally, the calculated (Δ*G*_ads_) decreased from −24 to −36 kJ mol^−1^ as alkyl chain length increased, indicating that nature of the adsorption began to exhibit characteristics of chemical adsorption, at least to some degree.^83^

**Fig. 9:**
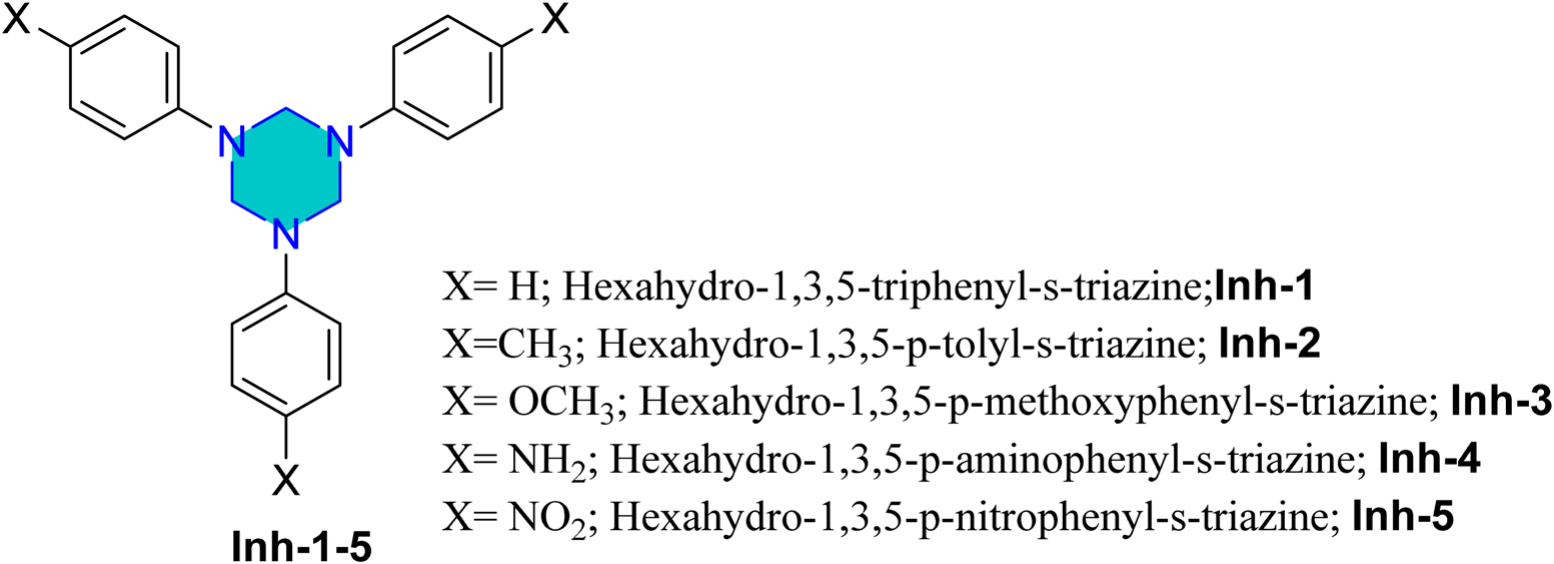
Structure of Inh-1, Inh-2, Inh-3, Inh-4, and Inh-5 as corrosion inhibitors for mild steel.

Shukla *et al.* reported the synthesis of five triazines Inh-1, Inh-2, Inh-3, Inh-4, and Inh-5 (Fig. 9).^84,85^ The corrosion inhibition performance of these compounds towards mild steel in 1 M HCl was evaluated using weight loss measurements in combination with and electrochemical techniques. The derivative Inh-4 showed the highest inhibition efficiency. Their adsorption followed the Langmuir isotherm, and the inhibition efficacy follow the order Inh-4 > Inh-3 > Inh-2 > Inh-1 > Inh-5.^84,85^

Later, Salman *et al.* investigated the corrosion inhibition performance of Inh-1, Inh-3, and Inh-5 (Fig. 9) for the N80 steel surface in 15% HCl. The results revealed that inhibition efficacy of 93.3% at an inhibitor concentration of 800 mg L^−1^, highlighting the strong protective capability of these derivatives under highly acidic conditions.^86^

**Fig. 10:**
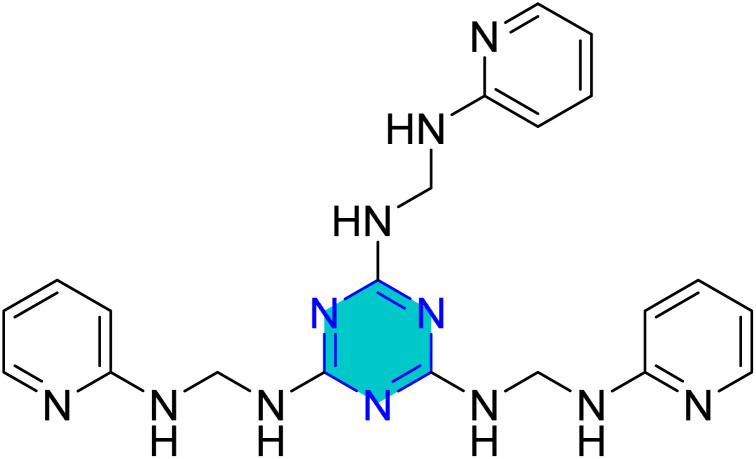
Structure of *N*_2_,*N*_4_,*N*_6_-tris((pyridin-2-ylamino)methyl)-1,3,5-triazine-2,4,6-triamine (INH).

Verma *et al.* reported the synthesis of a triazine derivative, INH (Fig. 10) *via* ultrasonic irradiation as example for the green technique and assessed its performance on mild steel in 1 M HCI.^87^ The adsorption of the inhibitor adhered to the Langmuir isotherm model and demonstrated a mixed type of inhibition performance with a predominance of cathodic effects.

**Fig. 11:**
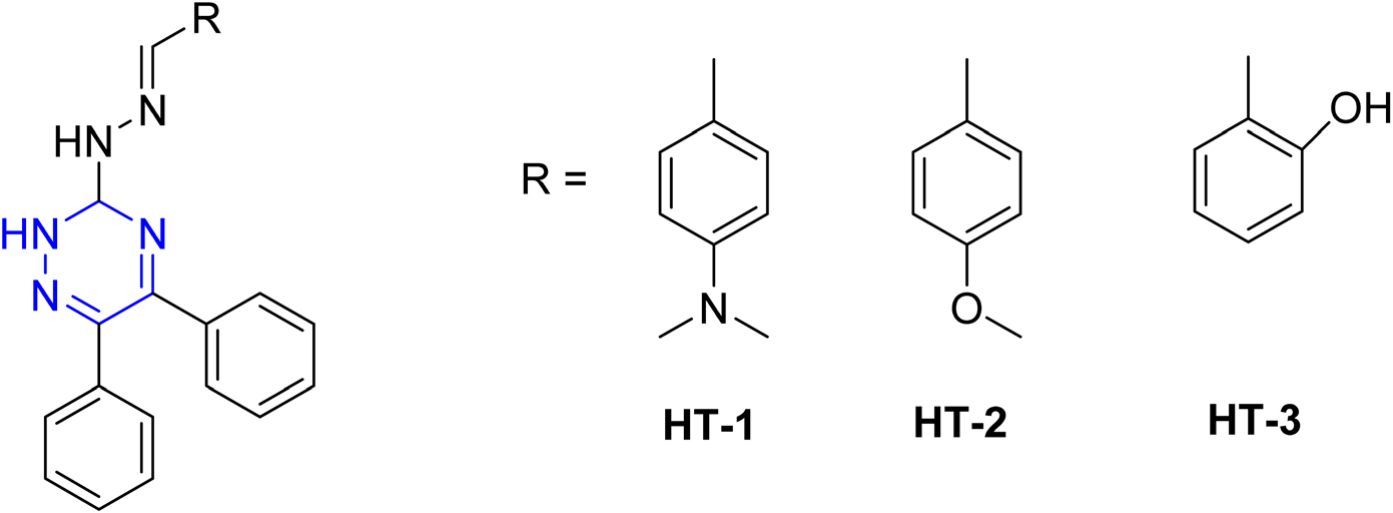
Structure of HT-1, HT-2, and HT-3 as corrosion inhibitors.

Singh *et al.* reported HT-1, HT-2, and HT-3 as triazine derivatives (Fig. 11). These triazines exhibited high inhibition efficiencies at low concentration (*e.g.*, 98.6% for HT-1 at 80 mg L^−1^). Their adsorption behavior obeyed the Langmuir isotherm and exhibited mixed-type inhibition behavior.^88^

**Fig. 12:**
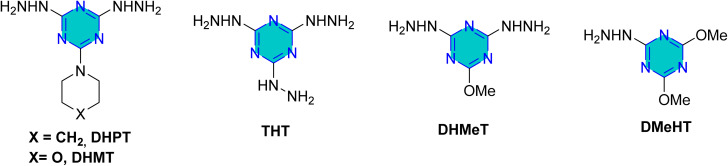
Structure of *s*-triazine hydrazine-based corrosion inhibitors in acidic media.

El-Faham group reported the synthesis of three hydrazino-*s*-triazine derivatives (Fig. 12), 2-hydrazino-4,6-dimethoxy-1,3,5-triazine (DMeHT), 2,4-dihydrazino-6-methoxy-1,3,5-triazine (DHMeT), and 2,4,6-trihydrazino-1,3,5-triazine (TH_3_) and examined their inhibition efficacy on steel in 1 M HCl.^89^ The results revealed that the number of hydrazine groups is crucial for corrosion inhibition; specifically, two hydrazine groups enhance electrostatic interactions with the negatively charged steel surface, which is influenced by chloride anion adsorption. Additionally, the methoxy group contributes to effective film formation on the steel surface due to the lone pairs of oxygen. However, increasing the number of hydrazine groups does not necessarily enhance efficiency, particularly at lower concentrations (25 ppm and 50 ppm). These derivatives were also examined for mild steel corrosion in 0.5 N H_2_SO_4_, reducing both metal dissolution and hydrogen evolution. The EIS results for all three inhibitors indicated a charge transfer-controlled process characterized by a single time constant. The adsorption of the inhibitors followed a mixed-type mechanism, adhering to the Langmuir isotherm. Notably, an efficiency of 95% was achieved at a low concentration of 25 mg L^−1^. Later, Prajila *et al.*^90^ reported the corrosion inhibition efficiency of three hydrazino-*s*-triazine derivatives, the authors reported that the corrosion efficacy increased with increase in inhibitor concentration and decreased with acid concentration and temperature. Also, the order of inhibition efficiency expected from the values of band energy obtained from UV-visible spectra in good agreement with the results obtained from weight loss and electrochemical techniques.^90^ This indicated that an increase in temperature accelerates the corrosion rate and promotes the desorption of organic molecules from the metal surface, leading to a decrease in inhibition efficiency.

El-Faham group also, reported 2,4-dihydrazino-6-morpholino-1,3,5-triaizne (DHMT) and 2,4-dihydrazino-6-piperidino-1,3,5-triaizne (DHPT) (Fig. 12)^91^ as excellent corrosion inhibitors in chloride-containing environments, with inhibition effectiveness increasing with concentration.^91^ The polarization studies classified both DHMT and DHPT as mixed-type inhibitors. The corrosion inhibition is attributed to the geometric blocking of active sites on the steel surface, leading to the formation of an insulating layer and significant changes in impedance response. Nyquist plots showed an increase in diameter with higher inhibitor concentrations, and the charge transfer resistance (*R*_ct_) values indicated better corrosion protection with increasing inhibitor concentration. The adsorption of DHMT and DHPT on steel occurs *via* a combination of physisorption and chemisorption. At lower concentrations (25 ppm and 75 ppm), DHMT performed better than DHPT, likely due to the presence of an oxygen atom that enhances film formation. At higher concentrations (150 ppm and 225 ppm), both compounds exhibited similar performance.^91^

**Fig. 13:**
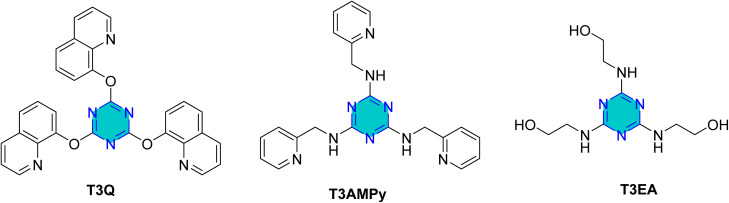
Structure of *sym*-trisubstituted *s*-triazine derivatives as corrosion inhibitors in acidic media.

In the same year, El-Faham group reported the synthesis of three *s*-triazine derivatives 2,4,6-tris(quinolin-8-yloxy)-1,3,5-triazine (T3Q), *N*_2_,*N*_4_,*N*_6_-tris(pyridin-2-ylmethyl)-1,3,5-triazine-2,4,6-triamine (T3AMPy), and 2,2,1,2,1,1-[(1,3,5-triazine-2,4,6-triyl)tris(azanediyl)]tris(ethan-1-ol) (T3EA) (Fig. 13),^92^ and evaluated for their ability to inhibit steel corrosion in hydrochloric acid solutions. The inhibition efficacy increased with concentration, reaching 98% at 250 ppm.^92^ The findings indicated that the adsorption of the compounds onto the steel surface well described by the Langmuir isotherm, indicating effective surface coverage. All three compounds acted as mixed-type inhibitors, suppressing both anodic and cathodic reactions. T3Q and T3AMPy exhibited superior inhibition effects compared to T3EA, and the presence of nitrogen atoms in the terminal groups of the inhibitors contributed positively to corrosion protection. Finally, the structural characteristics of the triazine derivatives, particularly the composition and nature of the side chains, significantly influence their corrosion inhibition performance.^92^

**Fig. 14:**
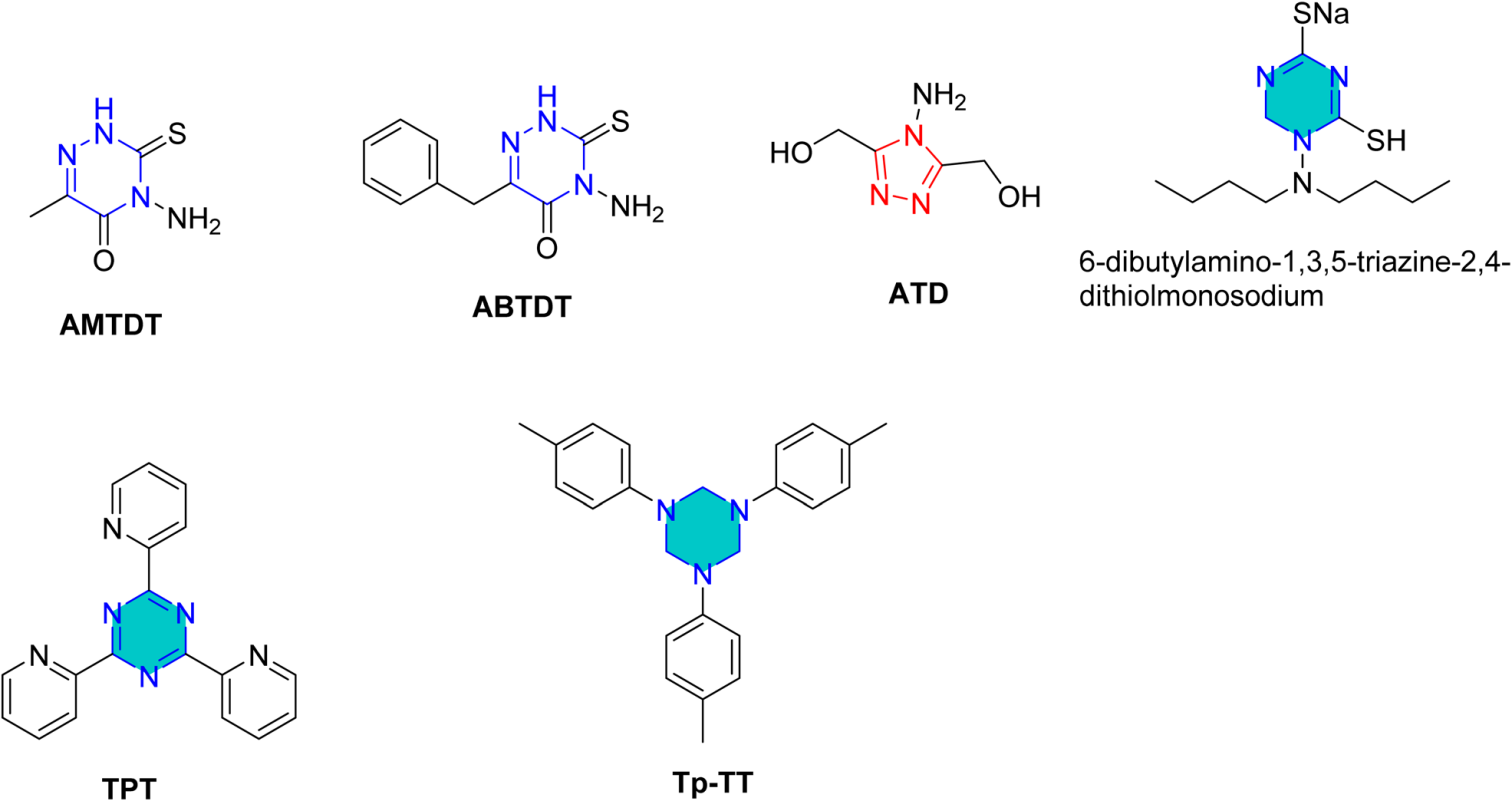
Structure of AMTDT, ABTDT, ATD, TPT, Tp-TT, and 6-dibutylamino-1,3,5-triazine-2,4-dithiolmonosodium as corrosion inhibitors in acid media.

Yadav *et al.* synthesized two novel triazine derivatives, 4-amino-6-methyl-3-thioxo-3,4-dihydro-1,2,4-triazin-5(2*H*)-one (AMTDT) and (4-amino-4*H*-1,2,4-triazole-3,5-diyl) dimethanol (ATD) as shown in Fig. 14, and evaluated them as corrosion inhibitors for N80 steel in 15% HCl.^93^ The study revealed that the inhibition efficacy increased with inhibitor concentration, while decreased with elevation in temperature. The same inhibitors were further examined for mild steel in 1 M HCl using electrochemical techniques and computational analyses.^94^ The results showed that the inhibitor adsorption adhered to the Langmuir isotherm and exhibited a mixed-type inhibition mechanism with predominantly cathodic character. Monte Carlo simulations revealed that the inhibitor AMTDT was located closer to the Fe(111) surface than the inhibitor ATD. At higher concentrations, both inhibitors exhibited comparable inhibition efficacies, however, at lower concentrations, AMTDT demonstrated significantly greater efficiencies than ATD.^94^

Soni *et al.* investigated the sodium salt of a phenyl triazine derivative, 3-hydroxy-3-(4-chlorophenyl-1-(4-sulphonato sodium salt)) phenyl triazene, HCST, as a corrosion inhibitor for brass in 0.5 M HNO_3_.^95^ The inhibition efficacy was found to increase with concentration inhibitor but decrease with rising temperature. Adsorption of HCST followed the Langmuir isotherm model, with the adsorption attributed primarily to the phenyl and SO_3_H functional groups.

El-Sayed *et al.* studied tin, indium, and tin–sodium alloys in 0.5 M HCl using a triazine derivative, 2,4,6-tris(2-pyridyl)-1,3,5-triazine (TPT) (Fig. 14).^96^ They suggested that the molecular structure of TPTZ enables it to act as a tridentate ligand, coordinating effectively with the metal surface. On tin, the inhibitor demonstrated chemisorption, while on indium the activation energy (*E*_a_) decreased with temperature, indicating a different mechanism of inhibition.

Furthermore, the 1,2,4-triazine precursors 4-amino-6-methyl-3-thioxo-3,4-dihydro-1,2,4-triazin-5(2*H*)-one (AMTDT) and 4-amino-6-benzyl-3-thioxo-3,4-dihydro-1,2,4-triazin-5(2*H*)-one (ABTDT) (Fig. 14) were assessed as inhibitors for aluminum in 1 N HNO_3_ using both electrochemical and computational approaches.^97^ It was observed that electron-donating substituents around the inhibitor molecules enhanced the local electron density, resulting in higher inhibition efficiency. Computational studies further revealed that the geometry of the inhibitor molecules changes upon adsorption, influencing their interaction with the metallic substrate.^97^

Zhao *et al.* reported the effect of 6-dibutylamino-1,3,5-triazine-2,4-dithiolmonosodium (Fig. 14) on stainless steel SUS304 in 1 M HCl using the electrochemical study.^98^ This inhibitor adhered mixed physical and chemical adsorption onto the stainless-steel surface. EIS studies showed the presence of inductive loops in the impedance spectra, while PDP results confirmed that the inhibitor act as a mixed-type inhibitor.

In addition to steel substrates, the triazine derivative Tp-TT (1,3,5-tri-*p*-tolyl-1,3,5-triazine) (Fig. 14) has been assessed for non-ferrous metals in acidic media.^99^ The results revealed that its adsorption was consistent with the Frumkin isotherm model and showed mixed-type adsorption.

**Fig. 15:**
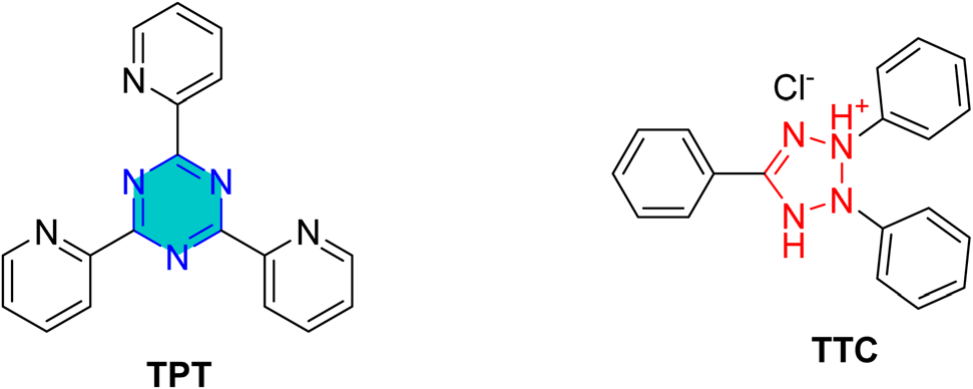
Tripyridyl and triphenyl *s*-triazine derives as corrosion inhibitors.

Xuehui *et al.*^100^ investigated the corrosion-inhibition performance of two organic derivatives, 2,3,5-triphenyl-2*H*-tetrazolium chloride (TTC) and 2,4,6-tri(2-pyridyl)-*s*-triazine (TPTZ) (Fig. 15) towards mild steel in 1 M HCl at room temperature. EIS results revealed that increasing the inhibitor concentration resulted in a significant increase in polarization resistance (*R*_p_) accompanied by decrease in double-layer capacitance (*C*_dl_), indicating effective adsorption of the inhibitor molecules at steel/solution interface. Potentiodynamic polarization (PDP) further demonstrated that both compounds acted as mixed-type inhibitors, suppressing anodic and cathodic processes without any change in corrosion mechanism. Quantum chemical analysis showed that enhanced efficiency correlated with the higher LUMO orbital density, greater molecular dipole, and a reduced HOMO–LUMO energy gap parameter that favor stronger interaction with metal surface.

Surface examination by scanning electron microscope (SEM) confirmed these findings, revealing that the addition of TTC and TPTZ significantly mitigated surface damage and protected the steel from aggressive acid attack.^100^

**Fig. 16:**
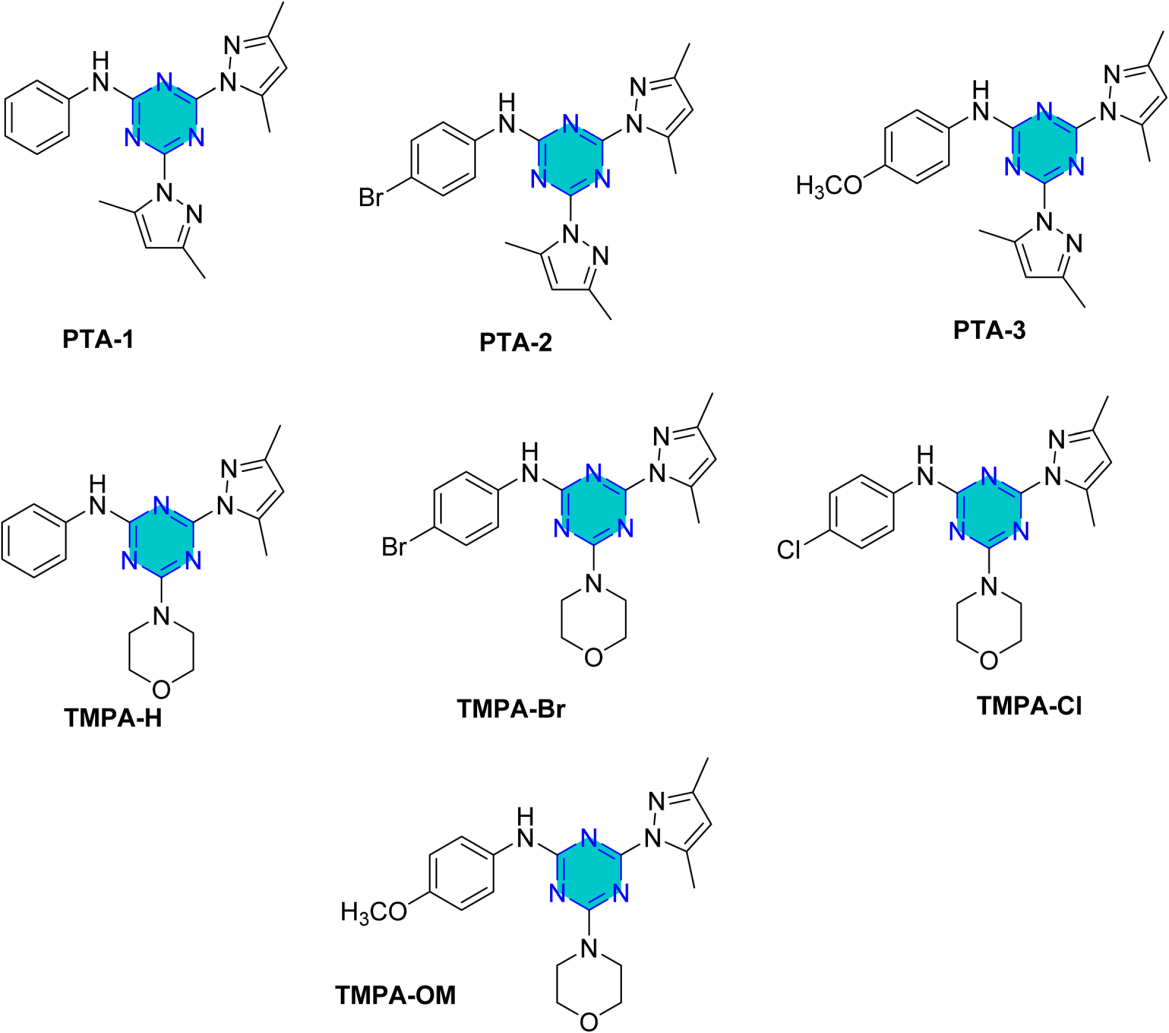
*s*-Triazine pyrazole derivatives reported corrosion inhibitors in acidic media.

El-Faham group^101^ reported the synthesis of three bis(3,5-dimethylpyrazolyl)-aniline-*s*-triazine derivatives, PTA-1 (4,6-bis(3,5-dimethyl-1*H*-pyrazol-1-yl)-*N*-phenyl-1,3,5-triazin-2-amine), PTA-2 (*N*-(4-bromophenyl)-4,6-bis(3,5-dimethyl-1*H*-pyrazol-1-yl)-1,3,5-triazin-2-amine), and PTA-3 (4,6-bis(3,5-dimethyl-1*H*-pyrazol-1-yl)-*N*-(4-methoxyphenyl)-1,3,5-triazin-2-amine), as shown in Fig. 16 and assessed them as corrosion inhibitors for carbon steel in 0.25 M H_2_SO_4_. Electrochemical impedance spectroscopy and potentiodynamic polarization revealed that all three compounds act as mixed-type inhibitors, with inhibition efficiency increasing alongside concentration due to higher polarization resistance, lower double-layer capacitance, and thicker protective film. PTA-2 and PTA-3, bearing –Br and –OCH_3_, showed superior inhibition efficiencies of 96.5% and 93.4% at 120 ppm, respectively, compared to unsubstituted PTA-1, which exhibited 79.0% at 175 ppm. Adsorption studies indicated that PTA-2 and PTA-3 follow the Langmuir isotherm, while PTA-1 follows the Frumkin model; in all cases, negative 
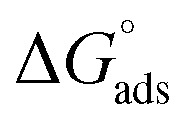
 values confirmed spontaneous adsorption *via* both physical and chemical interactions. Overall, both the triazine core and pyrazole units synergistically enhance anticorrosion performance, with substituent effects playing a key role in inhibitor efficiency.^101^

The same researchers reported the corrosion inhibition of C-steel in HCl solution using two *s*-triazine/morpholinoanilino-pyrazole derivatives,^102^ compound TMPA-H, 4-(3,5-dimethyl-1*H*-pyrazol-1-yl)-6-morpholino-*N*-phenyl-1,3,5-triazin-2-amine, and compound TMPA-Br, *N*-(4-bromophenyl)-4-(3,5-dimethyl-1*H*-pyrazol-1-yl)-6-morpholino-1,3,5-triazin-2-amine (Fig. 16). Electrochemical techniques, weight loss studies, and SEM analyses confirmed both compounds effectively reduced corrosion, with TMPA-Br showing higher efficiency (98.5% at 80 ppm *vs.* 97.8% at 100 ppm), attributed to its bromine substituent. Adsorption followed Langmuir behavior for TMPA-H and Temkin for TMPA-Br, both indicating spontaneous adsorption. SEM revealed smoother surfaces when inhibitors were used, while theoretical DFT analysis supported the superior performance of TMPA-Br through more favorable adsorption energy and molecular orbital properties. Overall, inhibition was achieved mainly by suppressing cathodic reactions, highlighting the significant role of structural substituents in enhancing steel protection.

Recently, in 2025, El-Faham group reported two triazine derivatives, *N*-(4-chlorophenyl)-4-(3,5-dimethyl-1*H*-pyrazol-1-yl)-6-morpholino-1,3,5-triazin-2-amine (TMPA-Cl) and 4-(3,5-dimethyl-1*H*-pyrazol-1-yl)-*N*-(4-methoxyphenyl)-6-morpholino-1,3,5-triazin-2-amine (TMPA-OCH_3_) (Fig. 16) and assessed them as corrosion inhibitors for C-steel in 1 M HCl using weight loss, impedance, potentiometric methods, SEM analysis, and computational approaches.^103^ Both hybrids acted as mixed-type inhibitors, providing high inhibition efficiencies of 96.5% for TMPA-Cl and 99.2% for TMPA-OCH_3_ at 100 ppm (0.26 mM), following Temkin and Langmuir adsorption isotherms with spontaneous adsorption. The superior performance of TMPA-OCH_3_, which incorporates a methoxy substituent, is attributed to the electron-donating effect of the OCH_3_ group that enhances surface adsorption and electron transfer, in contrast to the electron-withdrawing chlorine substituent in TMPA-Cl. These results indicate stronger, more stable adsorption of TMPA-OCH_3_ on the steel surface and demonstrate that the hybrid inhibitors, especially Morpho-OCH_3_ (TMPA-OCH_3_), achieve greater inhibition efficiency than many reported analogues due to their larger molecular size, reduced steric hindrance, electron-rich functional groups, and effective metal–inhibitor interaction.^103^

**Fig. 17:**
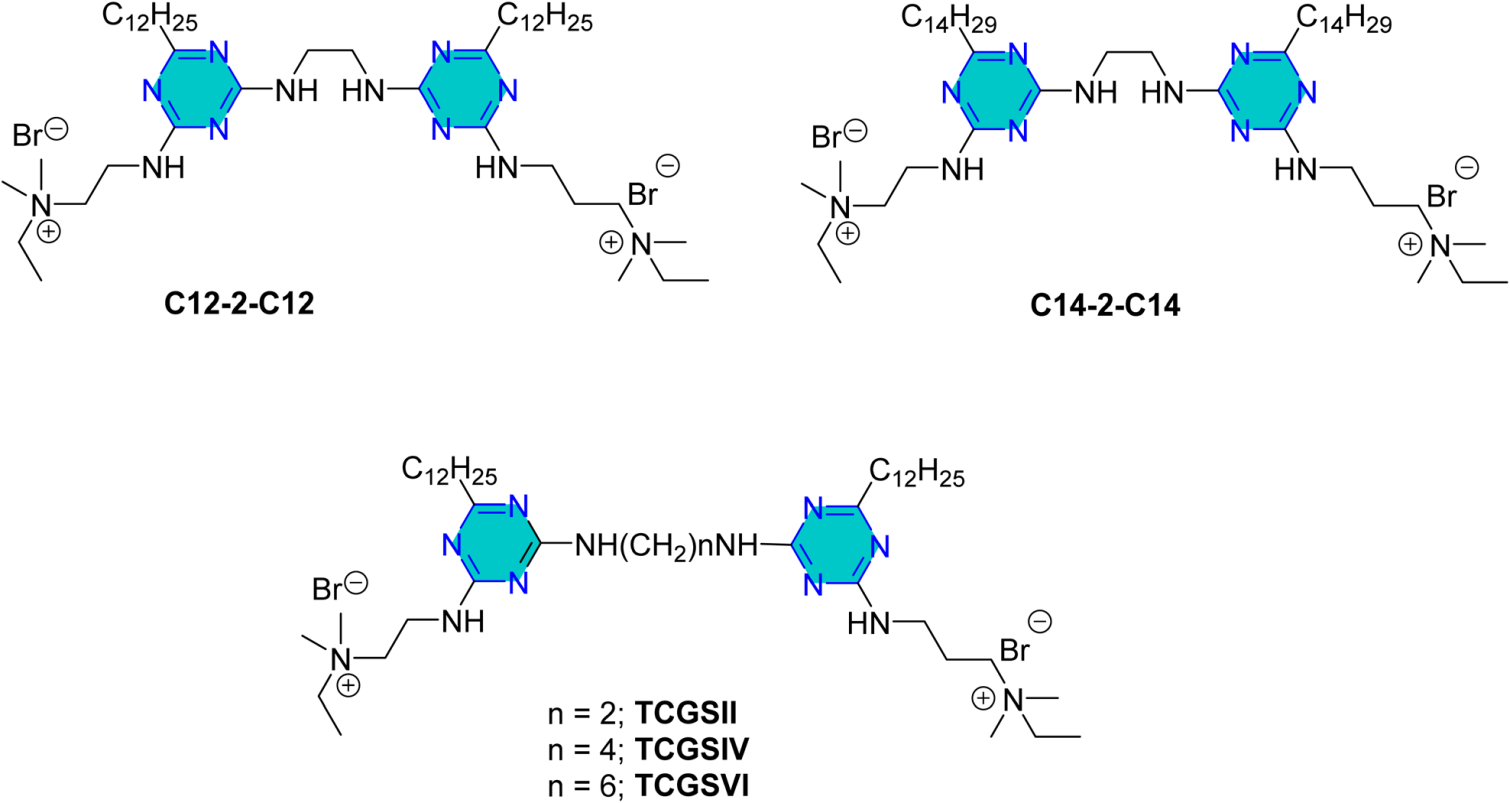
Structure of *s*-triazine derivatives C12-2-C12, C14-2-C14; and also TCGSII, TCGSIV, and TCGSVI as optional corrosion inhibitors.

In 2024, Gao *et al.*^104^ reported on the preparation and effectiveness of two novel triazine-based quaternary ammonium salt Gemini surfactants, C12-2-C12 and C14-2-C14 (Fig. 17), as potential corrosion inhibitors for carbon steel in environments containing sulfate-reducing bacteria (SRB). Their study demonstrated these surfactants' dual functions as corrosion inhibitors and bactericides. C14-2-C14, with its longer hydrophobic chain, consistently outperformed the other, showing lower minimum inhibitory concentrations (MICs) and higher corrosion inhibition rates. At a concentration of 0.2 mM, the inhibition rates were 93.23% for C14-2-C14 and 88.45% for C12-2-C12. Increasing surfactant concentrations led to enhanced corrosion inhibition due to improved adsorption capacity.^104^

Later, Yuan *et al.* in 2024,^105^ examined the corrosion inhibition performance of triazine-based cationic Gemini surfactants TCGSII, TCGSIV, and TCGSVI (Fig. 17) on Q235 carbon steel in 1 M HCl, utilizing both experimental and theoretical approaches. Their results indicated that these surfactants are effective corrosion inhibitors in acidic environments. Noticeably, they found that the length of the linking group significantly impacted their performance, with shorter lengths yielding better inhibition due to enhanced surface activity and adsorption. The corrosion inhibition rate decreased with longer linking groups, following the order: TCGSII > TCGSIV > TCGSVI. At a concentration of 0.2 mM, TCGSII achieved the highest corrosion inhibition rate of 98.03%.^105^

**Fig. 18:**
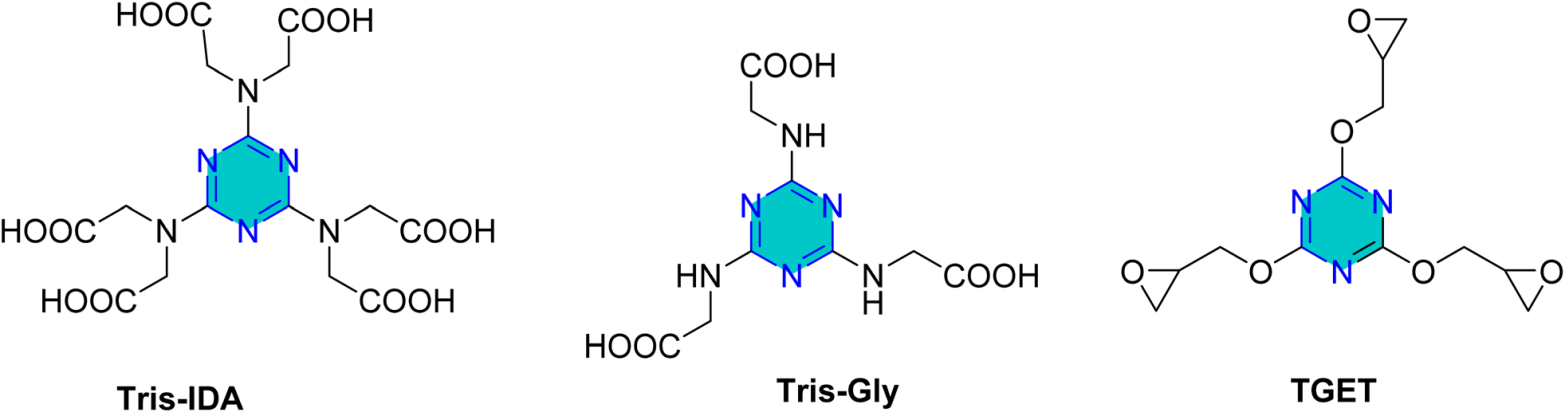
Structures of *s*-triazine amino acid derivatives (Tris-IDA and Tris-Gly) and triglycidyl ether triazine (TGET) as corrosion inhibitors.

Lgaz *et al.* in 2024,^106^ provided a comprehensive theoretical assessment of amino acid- and triazine-based corrosion inhibitors, Tris-IDA and Tris-Gly (Fig. 18), emphasizing the significance of electronic interactions and molecular structure in enhancing their performance. Their findings suggested that combining amino acids with triazine derivatives could be a promising strategy for developing effective corrosion inhibitors that form strong protective layers on metal surfaces.^106^

Rafik *et al.* in 2024,^107^ reported a detailed analysis of triglycidyl ether triazine (TGET) (Fig. 18), and evaluating its efficacy as a protective agent against corrosion of mild steel (C38) in a 1 M HCl. Through experimental and theoretical methods, they elucidated TGET's corrosion inhibition mechanisms. TGET exhibited exceptional corrosion inhibition efficacy for C38 steel in HCl, achieving a maximum of 94.77% at a concentration of 10^−3^ mol L^−1^. This high efficacy was attributed to the significant reduction in iron dissolution, attributed to the electron transfer from TGET's free electron pairs of heteroatoms (N and O) to the vacant d-orbitals of iron, leading to the formation of a protective film. The protonation of TGET in acidic conditions enhanced its interaction with the metal surface, thereby improving corrosion protection.^107^

**Fig. 19:**
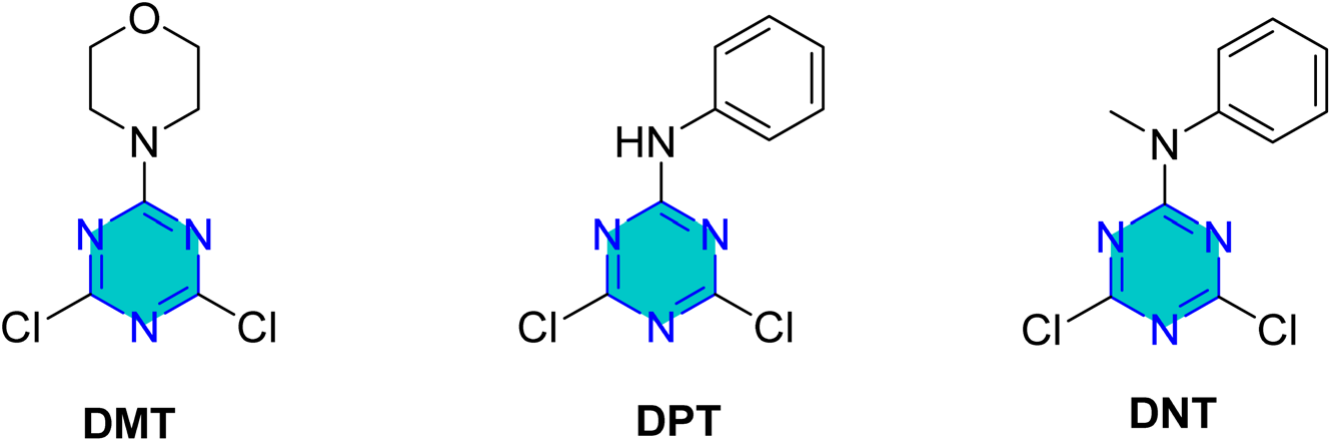
Structure of *s*-triazine derivatives (DMT, DPT, and DNT) as corrosion inhibitors in acidic media.

Very recently, Tshikhudo *et al.*^108^ investigated the corrosion inhibition capabilities of three synthesized substituted triazines (Fig. 19) 4,6-dichloro-2-morpholine-1,3,5-triazine (DMT), 4,6-dichloro-2-anilino-1,3,5-triazine (DPT), and 4,6-dichloro-*N*-methylanilino-1,3,5-triazine (DNT) on mild steel in 1 M HCl solution. All three compounds demonstrated effective corrosion inhibition, with the highest efficiency observed at a concentration of 0.005 M and at a temperature of 303 K. DMT achieved 93.87%, DPT reached 90.20%, and DNT showed 87.95%. The inhibition efficiency generally increased with higher inhibitor concentrations but decreased with rising temperatures. This effectiveness is attributed to their ability to form protective films *via* a combination of physical and chemical adsorption, involving electron donation from heteroatoms and π-electrons to the metal surface. These findings highlight the potential of these triazines as effective corrosion inhibitors.^108^

Collectively, Table 1 highlights a broad range of representative *s*-triazine derivatives that demonstrated excellent corrosion inhibition capabilities in several acidic media. Their performance can be tuned through structural modifications, positioning them as a promising class of compounds for various anti-corrosion applications.

**Table 1:** Representative *s*-triazine derivatives with excellent corrosion inhibition performance

Structure	Media	Evaluation means	Analysis	Corrosion efficiency	Ref.
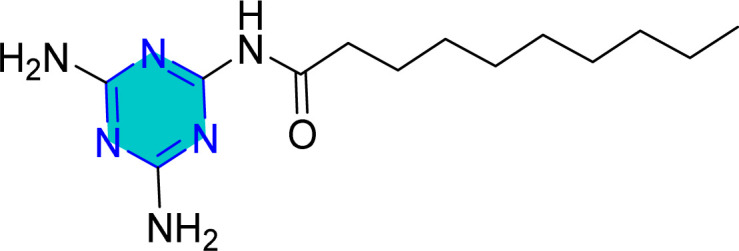	1 M HCl	Static weight loss; electrochemical impedance spectroscopy; potentiodynamic polarization	Mixed corrosion inhibitors	92.11% (1000 ppm)	109
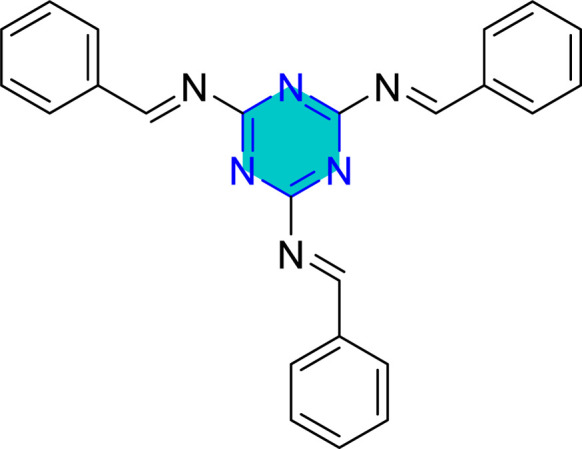	0.5 M HCl	Static weight loss; electrochemical impedance spectroscopy; potentiodynamic polarization	Mixed corrosion inhibitors	93.80% (100 ppm)	110
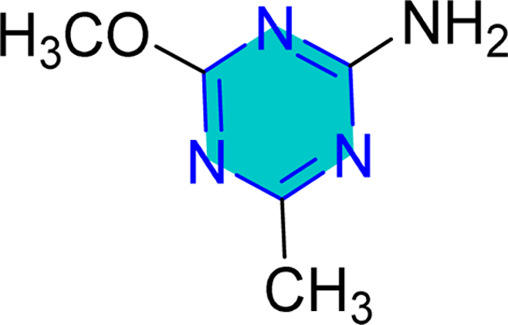	0.5 M HCl	Electrochemical impedance spectroscopy; potentiodynamic polarization	Mixed corrosion inhibitors	92.00% (140 ppm)	111
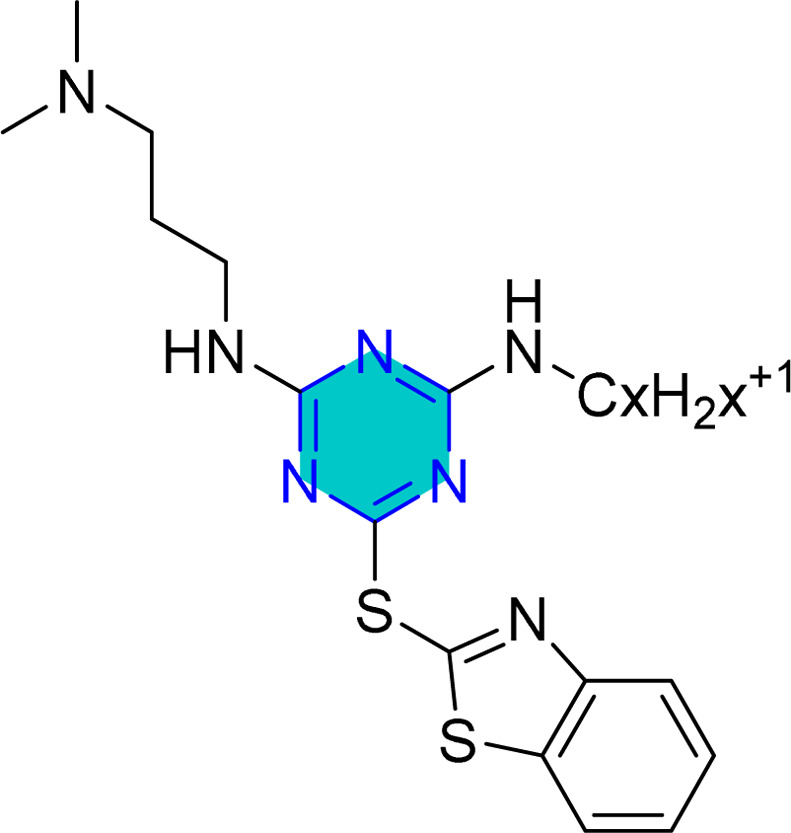	1 M HCl	Static weight loss; electrochemical impedance spectroscopy; potentiodynamic polarization	Mixed corrosion inhibitors	BTC6T 99% (435 ppm)	112
				BTC8T 99.3% (463 ppm)	
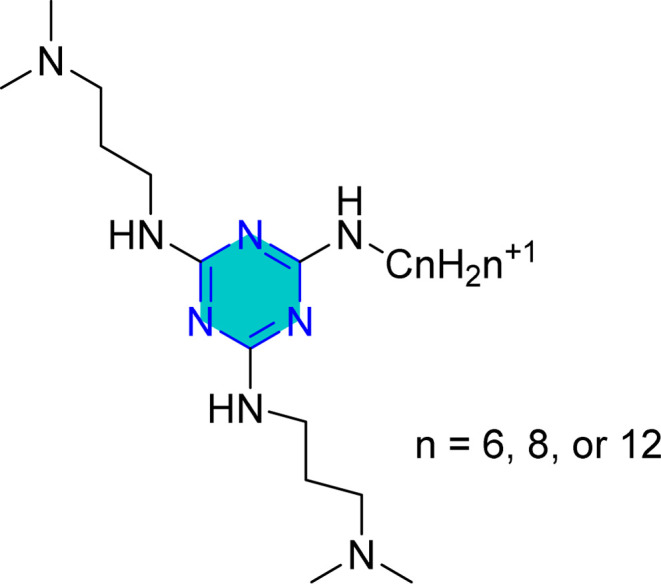	1 M HCl	Static weight loss; electrochemical impedance spectroscopy; potentiodynamic polarization	Mixed corrosion inhibitors	C6 95.5% (380 ppm)	113
				C8 94.8% (408 ppm)	
				C12 97.23% (464 ppm)	
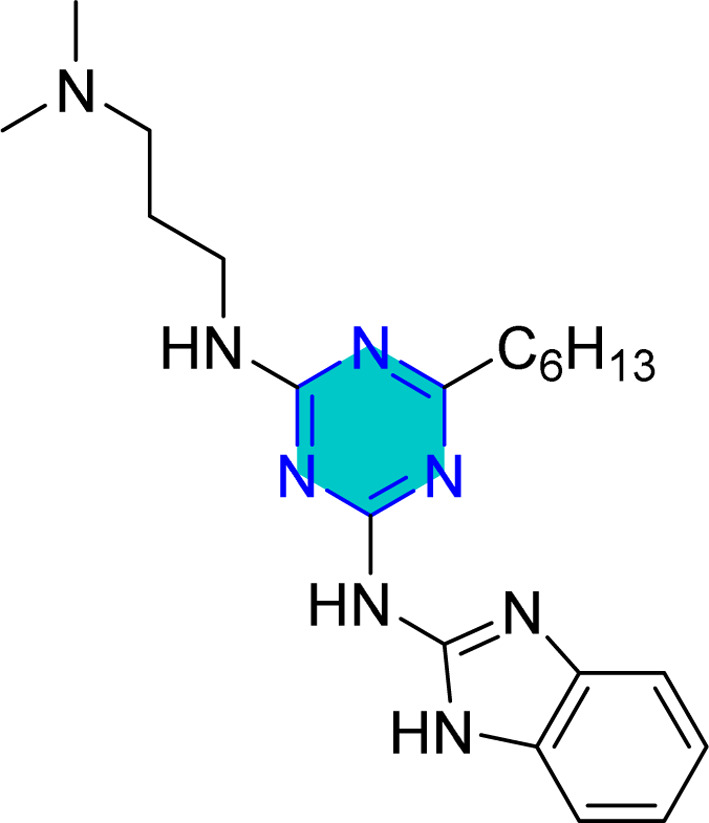	1 M HCl	Static weight loss; electrochemical impedance spectroscopy; potentiodynamic polarization	Mixed corrosion inhibitors	96.88% (411 ppm)	114

The majority of the reported corrosion inhibitors are low molecular weight organic compounds. Owing to their relatively small size, these molecules adsorb over limited metallic surface and often require comparatively higher concentration to achieve effective protection compared to polymeric inhibitors.^115–117^ In parallel, triazine derivatives have been extensively reported in an enormous number of applications.^118^ More recently, increasing attention has been directed towards triazine-based systems, due to the structural versatility and synthetic flexibility offered by the triazine core as a modular building block. In a relatively short timeframe, synthetic methodologies have been established to design triazine containing macromolecular and triazine-based dendritic materials. Emerging studies indicate that triazine-based dendritic materials exhibit promising performance as corrosion inhibitors and play an essential role in enhancing the corrosion protection efficiency.^16,119,120^

##### 1.1.3.2. Corrosion inhibition by triazine derivatives in neutral and salt media

Compared to the extensive literature addressing steel corrosion in acidic environments, there is a notable lack of studies focusing on triazine-based corrosion inhibitors for mild and carbon steel in neutral media. These neutral environments are relevant for applications such as sweet corrosion systems, heat exchangers, cooling water systems, and diverse water treatment processes.^37,121,122^

Conversely, a significant amount of research has focused on triazine derivatives as corrosion inhibitors for copper in neutral media, particularly in highly saline conditions like 3% to 3.5% NaCl. Heterocyclic corrosion inhibitors are frequently used in these systems due to their strong ability to coordinate with the metallic substrate.^123^

The compound (6-vinylbenzyl propyl)amino-1,3,5-triazine-2,4-dithiol demonstrated high corrosion resistance when deposited on copper plates.^124^ Zhou *et al.* investigated the inhibition performance of 6-aniline-1,3,5-triazine-2,4-dithiol (ATD) monosodium salt and benzotriazole (BTA) on copper surfaces in a 0.1 M Na_2_SO_4_ solution at pH 7.2 using electrochemical techniques. Their findings, supported by EIS, PDP, and surface-enhanced Raman studies, indicated that BTA outperformed ATD.^125^

Mori *et al.* examined the surface treatment of copper powders with triazine dithiol, revealing that the metal's response was significantly influenced by the type of functional group attached to the triazine dithiol, as well as experimental factors such as concentration, temperature, solvent, time, and oxide formation.^126^ Baba *et al.* conducted potentiostatic anodization using triazinedithiol (TDT) solutions, which showed that polymerization of TDT on copper substrates enhanced both hydrophobicity and corrosion resistance.^127^ Similarly, 6-dioctylamino-1,3,5-triazine-2,4-dithiol monosodium salt resulted in a protective, water-repellent copper surface.^128^

The compound *N*-(5,6-diphenyl-4,5-dihydro-[1,2,4]triazin-3-yl)-guanidine (NTG) achieved a remarkable efficiency of 99.8% at a 10^−2^ M concentration, with its adsorption behavior conforming to the Langmuir isotherm.^129^ Trithiocyanuric acid (TTCA) was assessed for its effects on copper corrosion in a 3% NaCl solution, employing weight loss, EIS, and PDP measurements, which indicated a mixed-type inhibition with a high efficacy of 95.3%.^123^

Self-assembled nanofilms of two triazine derivatives, AF17N and DAN, were formed on copper alloy surfaces, with their surface wettability and free energy evaluated.^130^ The AF17N film exhibited excellent hydrophobicity, achieving a contact angle of up to 124.10° and demonstrating higher corrosion resistance. Additional self-assembled monolayer, 2,4,6-trimercapto-1,3,5-triazine (TMTA), was examined on copper surfaces in 0.5 M NaCl, revealing through EIS and PDP measurements that TMTA acted primarily as a mixed-type corrosion inhibitor with a cathodic predominance.^131^

Mori *et al.* utilized 6-substituted-1,3,5-triazine-2,4-dithiols for the surface treatment of ultrafine magnetic iron powders, achieving enhanced corrosion inhibition as film thickness increased alongside improved water repellency.^132^ 5-(2-Hydroxyethyl)-1,3,5-triazine-2-thione (HOTAT) was applied to carbon steel in NH_4_Cl, exhibiting strong and exothermic adsorption behaviors, consistent with the Langmuir isotherm.^133^ The compound 6-dibutylamino-1,3,5-triazine-2,4-dithiol monosodium (DBN) displayed spontaneous and predominantly physical adsorption on brass in a 0.5 M NaCl solution, following the Langmuir isotherm and enhancing hydrophobicity.^134^ While, 6-diallylamino-1,3,5-triazine-2,4-dithiol (DAN) was electrochemically polymerized on pure aluminum surfaces in NaNO_2_, forming a nanofilm with excellent adhesion and anti-corrosion properties.^135^ DBN also showed spontaneous adsorption onto the brass surface, following the Langmuir isotherm, with increased inhibitor concentration correlating to a higher water contact angle, indicating improved hydrophobicity due to adsorbed inhibitors.

In summary as shown in Table 2, while triazine-based inhibitors have been less extensively studied for steel in neutral media/salt media, they have demonstrated significant promise and effectiveness, particularly for copper and other non-ferrous metals, often forming protective films through various adsorption mechanisms.

**Table 2:** Triazine-derived corrosion inhibitors in neutral and salt media

Inhibitor	Metal/medium	Techniques	IE%	Nature of adsorption	Reference
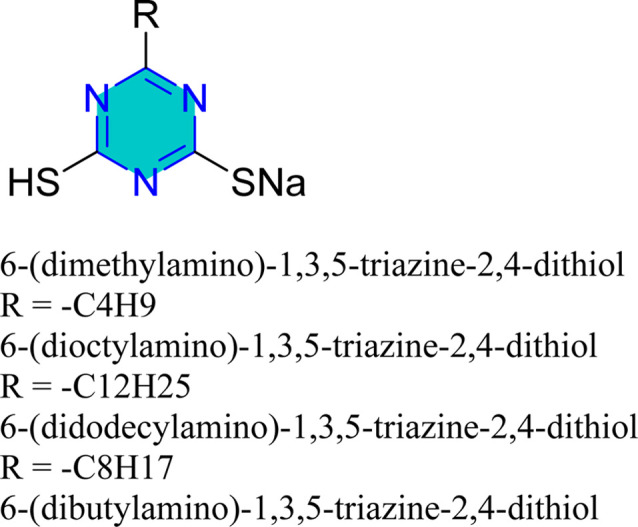	Cu powder/moist air	Humidity, temperature tests	—	—	126
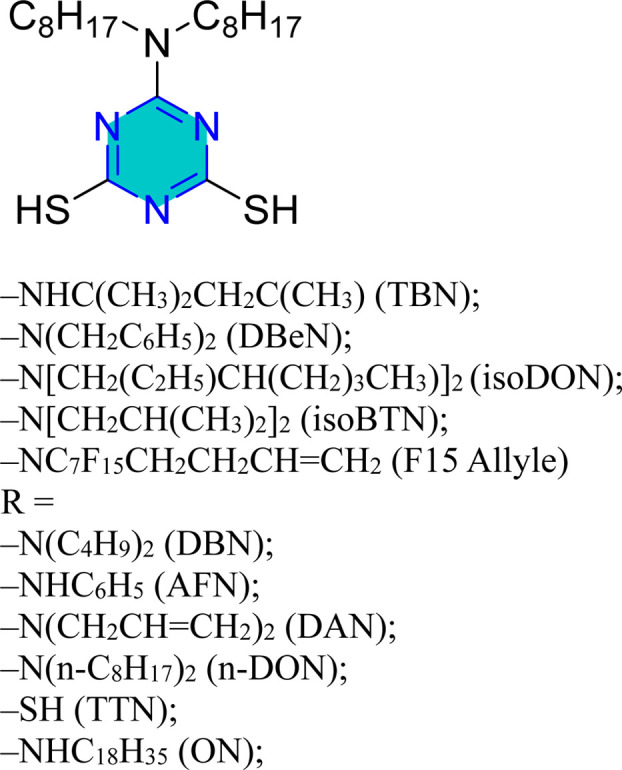	Cu/3% NaCl	WCA; FTIR, GPC	71% (DBN)	—	127
			−31.5% (AFN)		
			57.9 (DAN)		
			89.4 (*n*-DON)		
			63.1% (TTN)		
			89.4% (ON)		
			−7.9% (TBN)		
			76.3% (DBeN)		
			89.4 (isoDON)		
			57.9 (isoBtN)		
			73.7 (F15-Allyle)		
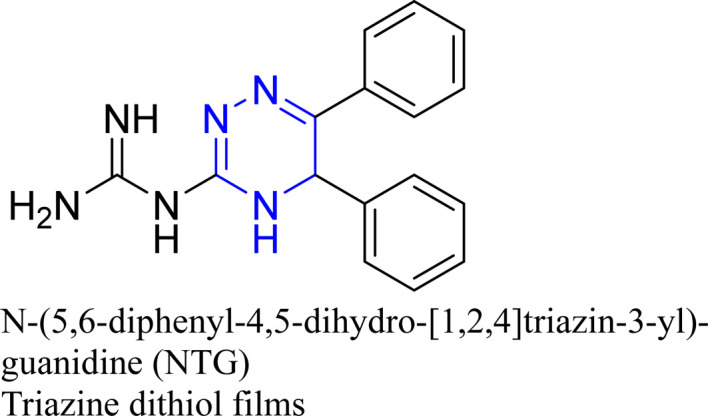	Cu/3% NaCl	WCA, XPS	83.2%	—	128
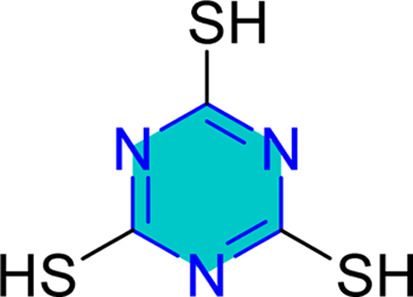	Cu/3% NaCl	WL; EIS, EFM, PDP; PM3	99.47%	Langmuir, mixed type	129
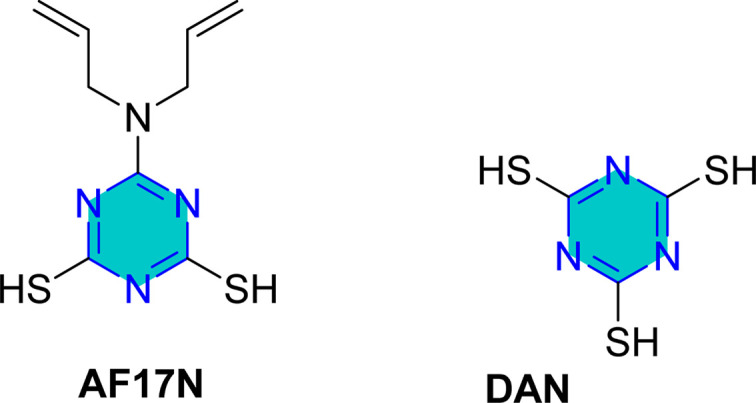	Cu/3.5% NaCl	WCA; LPR, PDP, CV; SE	97.4% (AF17N)	—	130
			89.4% (DAN)		
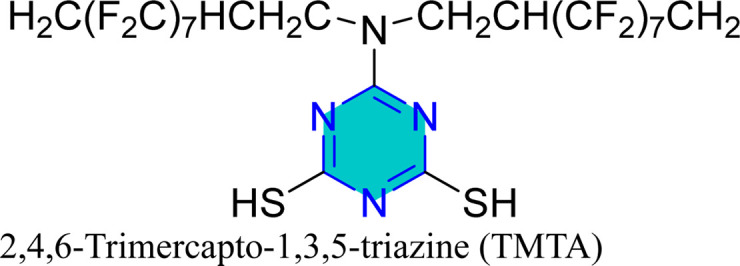	Cu/0.5 M NaCl	EIS, PDP; SEM; DFT	93.9%	—	131
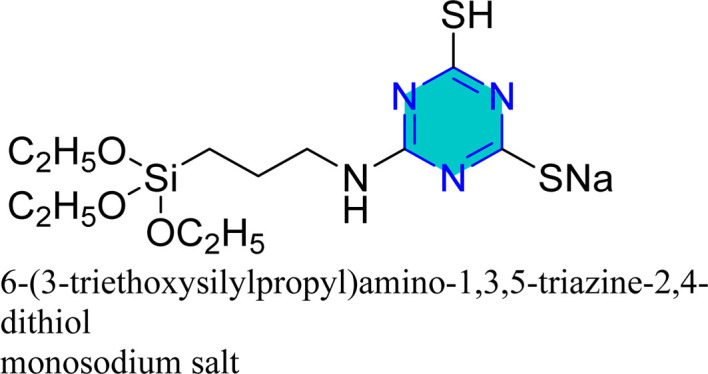	Cu/3.5% NaCl	EIS, PDP, CV; WCA, XPS	95.59%	Mixed type	136
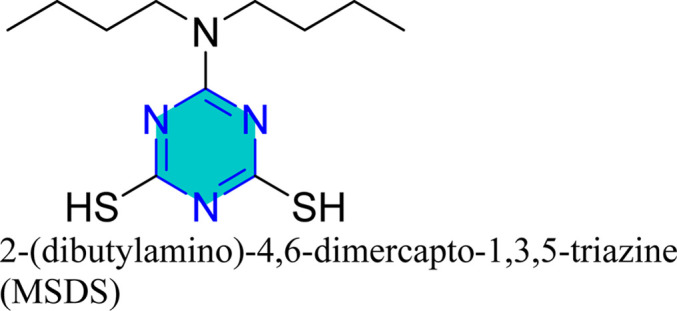	Cu/3.5% NaCl	EIS, PDP; SEM, AFM, WCA; DFT, MD	97.91%	—	137
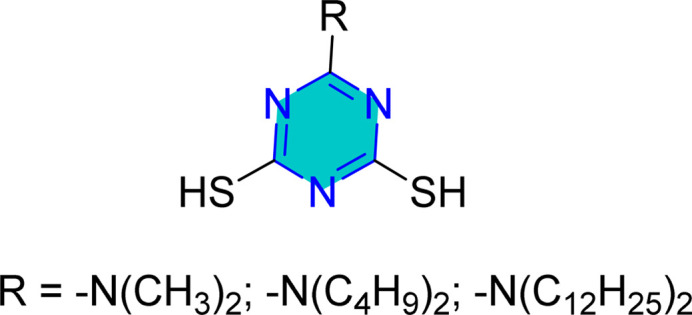	Magnetic Fe powder/moist air	Humidity, temperature tests; XRD	—	—	132
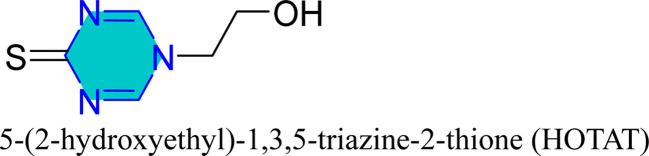	Carbon steel/NH_4_Cl	WL; EIS, PDP; SEM	82%	Langmuir, mixed type	133
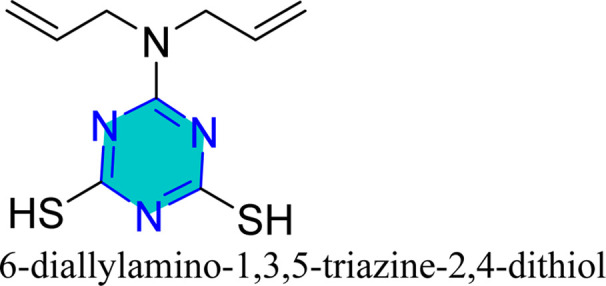	Al/0.15 M NaNO_2_	CV; SEM, XPS	—	—	135

### 1.2. Impact of molecular structure on triazine corrosion inhibitors

The primary goal in developing efficient corrosion inhibitors is the rational design of organic molecules that exhibit extensive coverage of the metal surface, while exhibiting favorable wetting properties and adsorbing behavior. A compound that meets these criteria can be considered an effective corrosion inhibitor.^138,139^

At concentrations below the optimal dosage, inhibitor molecules tend to adsorb onto the metal substrate in a flat or horizontal orientation, maximizing surface coverage. Conversely, at concentrations above the optimum, these inhibitors can adopt vertical and parallel orientations, further enhancing adsorption on the metal surface. Larger inhibitor molecules, such as oligomers and polymers, are more effective at lower doses, while smaller molecules necessitate higher concentrations to achieve similar levels of coverage.

Several structural features influence the adsorption of corrosion inhibitors, including the presence of heteroatoms, stereochemistry, and the size of aromatic rings. Computational techniques can help correlate these structural features with inhibitor performance, particularly in the case of triazine-based inhibitors.^140,141^ Isin *et al.*^142^ evaluated *s*-triazines using multiple linear regression (MLR) to analyze the effects of substituents and quantum chemical parameters. Parameters such as EHOMO, ELUMO, electronegativity, and Δ*N* displayed strong correlations with experimentally determined corrosion inhibition efficiencies. The study also explored various substituents like –NH_2_, –OCH_3_, –CH_3_, –H, and –NO_2_, noting trends in electron-withdrawing properties. The influence of the electron-donating and electron-withdrawing substituents on inhibition performance has been comprehensively discussed by Verma *et al.*^4^ Al-Sabagh *et al.*^82^ investigated the impact of ethylene oxide (EtO–) groups on triazine derivatives, finding that while log *P* values decreased with increasing EtO– groups, quantum chemical parameters like Δ*N* and polarizability increased. This suggests that modifying pendant groups could enhance solubility and improve inhibitor efficacy.^85^

Computational studies involving non-ionic surfactants based on *s*-triazine supported experimental observations, revealing the role of N and O atoms in the adsorption process at the metal surface. In a density functional theory (DFT) study performed at the B3LYP/6-31++G(d,p) level, Isin and Karakus evaluated a series of *s*-triazines, HTT, HPTT, HPMeT, HPAT, and HPNT for mild steel corrosion in 1 M HCl. Their results demonstrated a clear correlation between substituent electronic effects and inhibition efficiency. Specifically, derivatives bearing electron-donating groups exhibited superior corrosion protection compared to those containing electron-accepting ones.^142^ The inhibition efficiency followed the order: –NH_2_ > –OCH_3_ > –CH_3_ > –H > –NO_2_. This trend underscores the enhancing role of electron-donating substituents in facilitating stronger adsorption interaction with metal surface, whereas electron-withdrawing groups were associated with comparatively reduced inhibitory performance.

Monte Carlo (MC) studies indicated a parallel adsorption orientation of inhibitors on the Fe(110) surface, correlating with experimental findings. Al-Sabagh *et al.*^82^ evaluated hexahydro-1,3,5-triazine derivatives, confirming that major adsorption sites were the N atoms within the triazine rings and polar functional groups. Monte Carlo simulations validated these trends in both vacuum and aqueous environments.^143^

A study on a guanidine-functionalized triazine revealed that the molecule NTG could adsorb on copper substrates by sharing electrons with N atoms, forming Cu–N coordinate bonds and π-electron interactions. Additionally, molecular dynamics simulations on tri-thiocyanuric acid indicated that the three sulfur atoms of the inhibitor played a crucial role in adsorption.^124,130^ A DFT study of a self-assembled film of 2-(dibutylamino)-4,6-dimercapto-1,3,5-triazine on copper showed high dipole moments, proposing that this characteristic enhances adsorption, although the correlation between dipole moment and inhibition behavior remains debated in the literature.^16,76^

Density Functional Theory (DFT) calculations for the *s*-triazine pyrazole derivatives (TMPA-H, TMPA-Cl, TMPA-Br, TMPA-OCH_3_, PTA-2, and PTA-3, Fig. 16) reported by El-Faham group^101–103^ revealed that TMPA-Br exhibited superior inhibition efficiency due to its favorable electronic structure, characterized by a higher HOMO energy and a lower LUMO energy, resulting in a smaller energy gap. Similarly, TMPA-OCH_3_ showed better performance than TMPA-Cl because of its electron-donating methoxy group, which improved reactivity. For the bis(dimethylpyrazolyl)-aniline-*s*-triazine derivatives, PTA-2 and PTA-3 demonstrated enhanced corrosion inhibition by facilitating stronger adsorption through both physical and chemical mechanisms, aided by electron-donating groups.

In summary, the effectiveness of corrosion inhibitors is significantly influenced by their molecular structure and orientation during adsorption. Computational techniques can provide valuable insights into optimizing these inhibitors by correlating structural features with performance. Continued research in this field, particularly focusing on the roles of various substituents and molecular interactions, is essential for developing more effective corrosion inhibitors for industrial applications.

### 1.3. Monte Carlo simulation insights into corrosion inhibition

Monte Carlo (MC) simulations have been extensively employed to investigate the interaction of triazine derivatives with steel surface, with the objective of clarifying the adsorption mechanism at the molecular level. These simulations were specifically designed to analyze binding configuration, adsorption energies, and the overall interaction patterns between the inhibitor molecules and the metal substrate. Through MC modeling, detailed insights into the orientation, surface coverage, and strength of interaction of the inhibitors were obtained. In addition, MC simulations, combined with quantum chemical calculations, help illustrate the optimum configuration of triazine molecules adsorbed onto the metal surface and compute their interaction energies, which are essential for understanding inhibition performance. Table 3 compared the three computational methods that give insights into corrosion inhibition. Those are density functional theory (DFT),^144^ Monte Carlo (MC),^145,146^ and molecular dynamics (MD).^147^

**Table 3:** Summary for the computational studies insights into corrosion inhibition

Method	Primary purpose	Typical outputs	Strengths and limitations	Ref.
Density functional theory (DFT)	Quantum reactivity of isolated molecules and small slabs	HOMO/LUMO energies, Δ*E*_gap_, Δ*N*, Fukui indices, ESP, charge analyses	High accuracy for electronic descriptors but costly for large/periodic systems	144
Monte Carlo (MC)	Sampling adsorption ensembles and locating low-energy adsorbate configurations	Adsorption energy distributions, probable orientations, coverage statistics	Efficient sampling of configurational space; does not provide time evolution	145 and 146
Molecular dynamics (MD)	Time evolution, solvent and temperature effects, film formation	Trajectories, diffusion coefficients, dynamic adsorption behavior	Captures kinetics and solvent-mediated rearrangements but requires longer runs and force-field quality checks	147

**Fig. 20:**
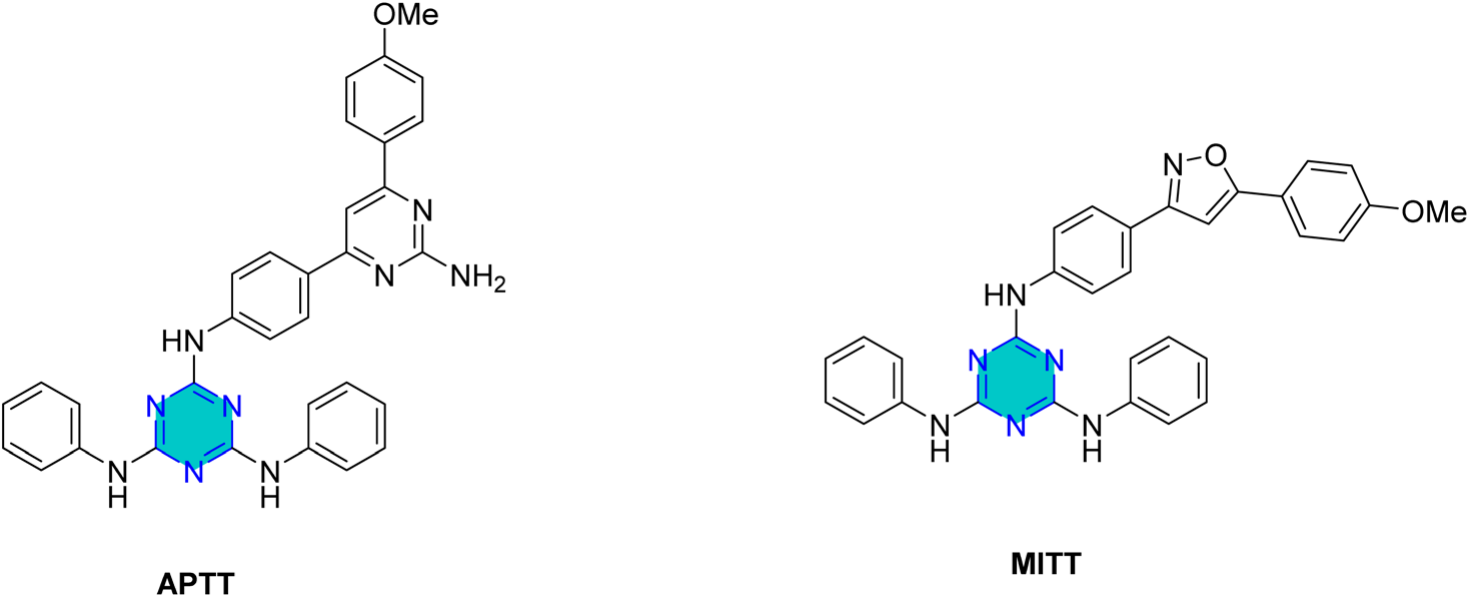
Structure of APTT and MITT.

Two recent studies investigated the efficacy of different synthesized triazine derivatives as corrosion inhibitors for steel in acidic environments.^148^ One study by Yadav *et al.*^93^ focused on two triazine derivatives, APTT and MITT (Fig. 20), for N80 steel in 15% HCl, while other triazines were examined by El-Faham group^101–103^ (Table 4). Both studies found that inhibition efficiency increased with inhibitor concentration, and the compounds with weak electron-withdrawing or electron-donating groups such as –Br, and OCH_3_ generally performed better than those with electron-withdrawing or less-donating groups –Cl. The inhibitors in both studies were classified as mixed type, affecting both anodic and cathodic reactions.^93,101–103^

**Table 4:** MC simulation results computed under corrosive medium for the adsorption of the pyrazolo-*s*-triazine molecules on steel^101–103^

Structure	Adsorption energy^a^	Rigid adsorption energy^a^	Deformation energy^a^	Inhibitor d*E*_ad_/d*N*_i_	Chloride d*E*_ad_/d*N*_i_	Hydronium d*E*_ad_/d*N*_i_	Water d*E*_ad_/d*N*_i_
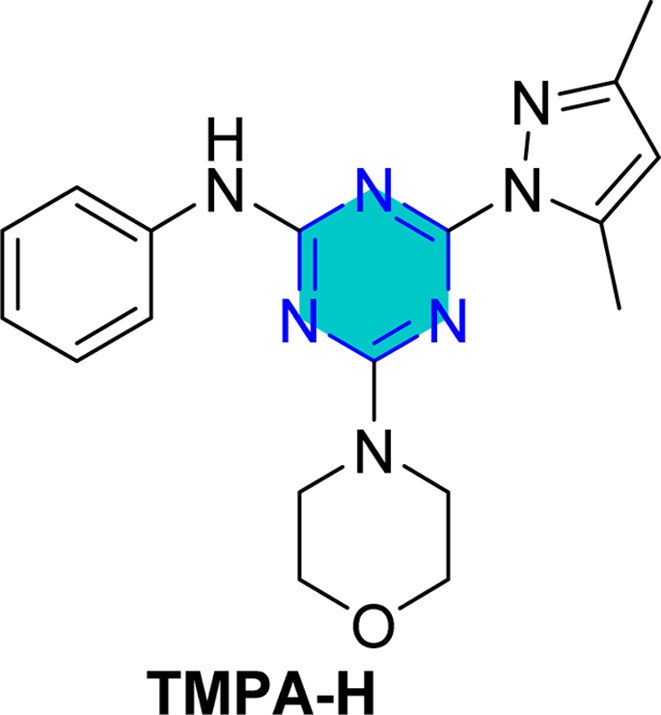	−33728.94	−18744.24	−14984.74	−1554.94	−601.45	−263.00	−77.53
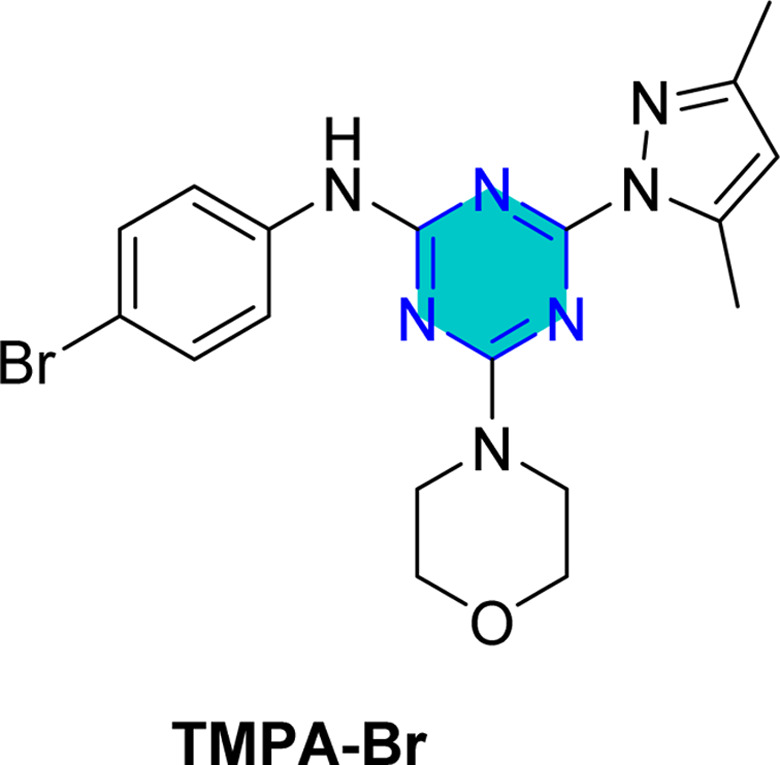	−33825.84	−18860.64	−14965.21	−1593.73	−591.62	−252.30	−76.86
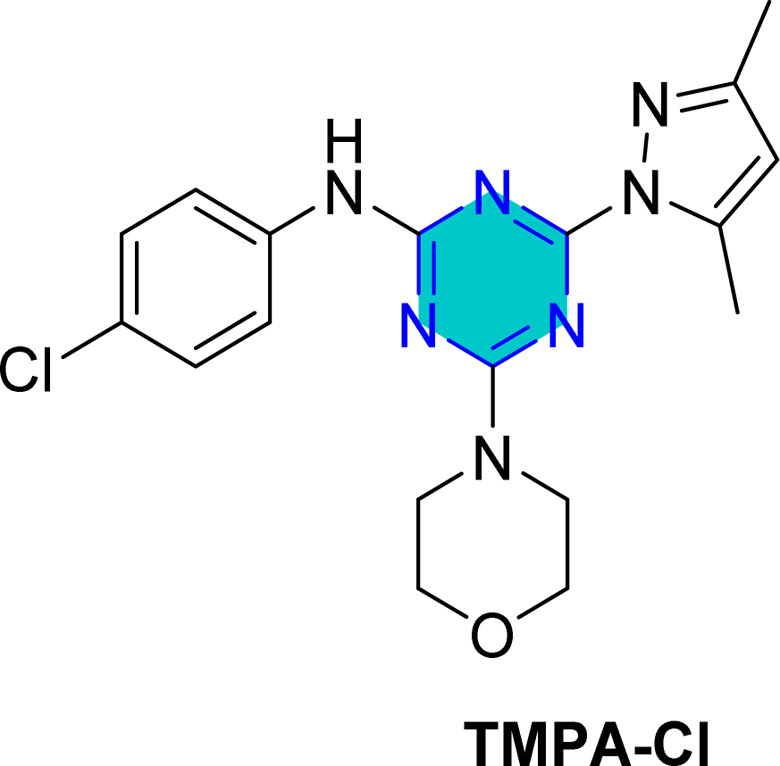	−33 720.03	−18 759.76	−14 960.31	−1458.71	−606.93	−262.29	−78.49
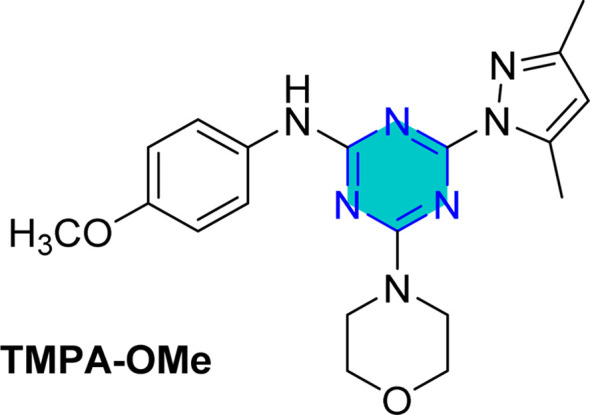	−33867.64	−18 891.60	−14 976.04	−1563.60	−602.24	−257.99	−76.02
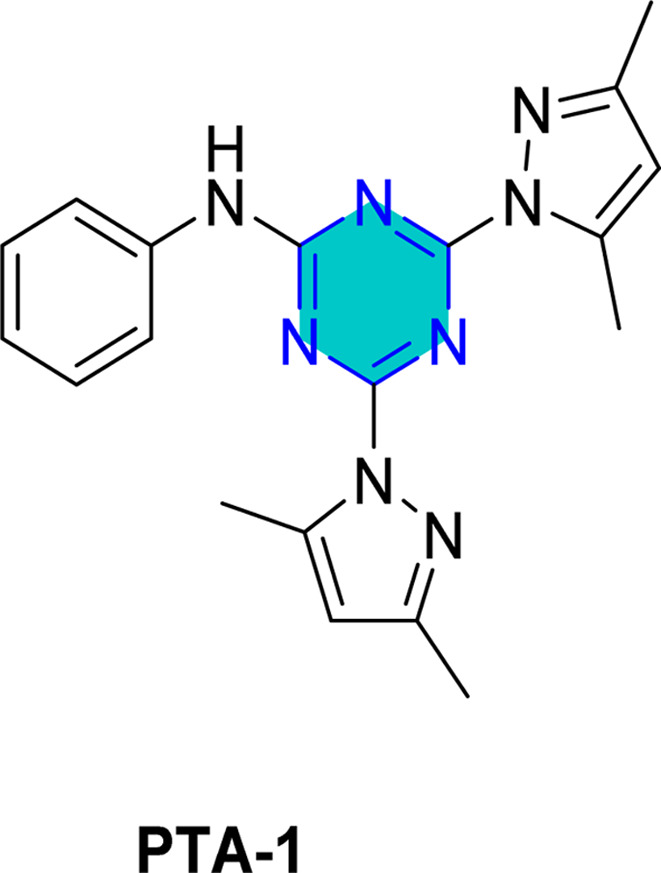	−7388.02	−5674.22	−1713.81	−1793.81	−507.64	−346.14	−72.13
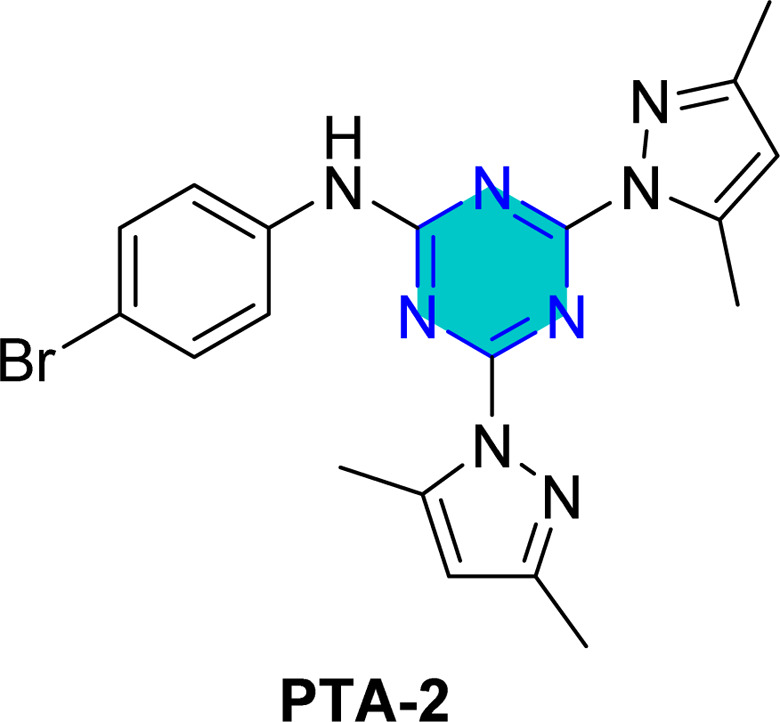	−8838.03	−7055.19	−1782.84	−1839.83	−510.91	−342.71	−73.09
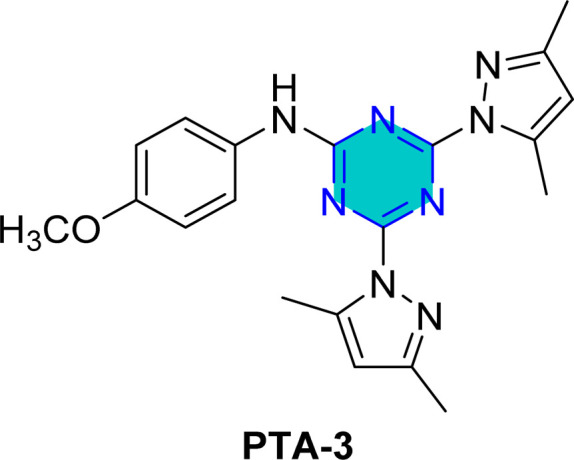	−8896.69	−7055.60	−1841.09	−1826.82	−510.53	−343.00	−72.89

a Energy (kJ mol^−1^).

The adsorption of APTT and MITT inhibitors on the N80 steel surface followed the Langmuir adsorption isotherm. The calculated Gibbs free energy of adsorption (Δ*G*_ads_) values ranged from −38.6 to −41.6 kJ mol^−1^ for APTT and −38.7 to −41.5 kJ mol^−1^ for MITT, suggesting a mechanism involving both physisorption and chemisorption.^93^ The inhibition efficiency of both APTT and MITT decreased as the temperature increased from 303 K to 333 K. This is attributed to the increased rate of desorption of the inhibitor molecules from the steel surface at higher temperatures. APTT, which has a pyrimidine ring, consistently showed better performance than MITT, which has an isoxazole ring.^93^

Recently, El-Faham group reported two different series of mono-pyrazolo-*s*-triazine (TMPA-H, TMPA-Cl, TMPA-Br, and TMPA-OMe)^102,103^ and bispyrazolo-*s*-triazine (PAT-1, PAT-2, and PAT-3) as shown in Table 4.^101^

For the mono-pyrazolo series (TMPA-H, TMPA-Cl, TMPA-Br, and TMPA-OMe) results highlight the superior performance of the bromo- and methoxy derivatives over the chloro- and the unsubstituted derivatives. The weak electron-withdrawing or electron-donating groups (Br- and methoxy group) enhances its inhibition efficiency compared to the electron-withdrawing chlorine group and the unsubstituted one (Table 4).^102,103^

Similarly, the rigid adsorption energy for molecules for methoxy was more negative than that of the chloro, suggesting better protection efficiency for the methoxy derivative.^103^ The energy associated with the metal/adsorbate arrangement, excluding adsorbed water molecules, was higher for inhibitor for the electron donating methoxy and Br than for unsubstituted and the chloro inhibitor.^102,103^ This implies superior adsorption of OMe and Br– compared to H– and Cl–.^102^

For the bispyrazolo-*s*-triazine (PTA-1-3) reported by El-Faham group,^101^ the MC simulates were employed to elucidate the interaction behavior between the inhibitors and the steel surface under corrosive medium.^101,102^ These simulations were conducted using the adsorption locator module, which enable the identification of the most favorable adsorption configurations and provided insight into binding characteristics of the inhibitors at metal surface.^102^Table 4 summarize the adsorption energies derived from Monte Carlo (MC) simulation under corrosive medium. The results from the two derivatives PTA-2 and PTA-3 molecules exhibited higher negative adsorption energy values compared to PTA-1. This indicates energetically favorable adsorption and the formation of a stable adsorbed film that protects the steel.^102^ The more negative adsorption energy values for PTA-2 and PTA-3 suggest stronger binding to the steel surface, highlighting their superior potential as corrosion inhibitors. The Δ*E*_ad_/Δ*N*_i_ values for PTA-2 and PTA-3 were higher than that of PTA-1, indicating stronger adsorption for PTA-2 and PTA-3.^101^ This aligns with experimental inhibition efficiency results.^101^ The Δ*E*_ad_/Δ*N*_i_ values for water molecules, sulfate ions, and hydronium ions were lower than those for the inhibitor molecules, suggesting that the inhibitors robustly adsorbed and displaced these species, forming a protective layer on the steel surface.^143^

In summary, both sets of triazine derivatives proved to be effective corrosion inhibitors. The compounds with stronger electron-donating substituents (APTT and Morpho-OCH_3_) exhibited higher inhibition efficiencies. The adsorption mechanisms for all inhibitors involved a combination of physical and chemical processes, and they all functioned as mixed-type inhibitors. The *s*-triazine-anilino-morpholino-pyrazolyl hybrids, particularly Morpho-OCH_3_, demonstrated exceptionally high efficiency at lower concentrations compared to APTT and MITT.

Competitive adsorption and displacement of water at the metal interface: all simulations were performed with explicit water and representative ionic species to mimic the corrosive aqueous environment. Under these conditions, inhibitor molecules approach the hydrated steel surface and compete with the pre-adsorbed water molecules and solvated ions for surface sites. The process can be described qualitatively as competitive adsorption, where adsorption of the inhibitor leads to partial or complete displacement of the hydration layer and formation of a more strongly bound inhibitor film. Such displacement reduces direct contact between aggressive species (*e.g.*, Cl^−^, H^+^) and the metal, thereby contributing to the experimentally observed inhibition efficiency. Atomistic simulations and prior studies indicate that hydrophobic moieties promote removal of structured water near the surface while polar/N-containing headgroups form specific interactions with surface iron atoms or oxide sites, stabilizing the adsorbed layer.

### 1.4. Adsorption behavior and inhibition mechanism of triazines derivatives

Corrosion onto a metal surface exposed to an electrolyte involves two primary electrochemical reactions: the dissolution of the metal at the anode and the evolution of hydrogen at the cathode (in acidic pH) or oxygen reduction (in near-neutral pH).^112^ When a corrosion inhibitor, such as a triazine derivative, is added to an acidic corrosive electrolyte, it can undergo protonation and deprotonation as indicated in the following ways.^149^Inh_neutral_ ⇌ Inh_protonated_

**Fig. 21:**
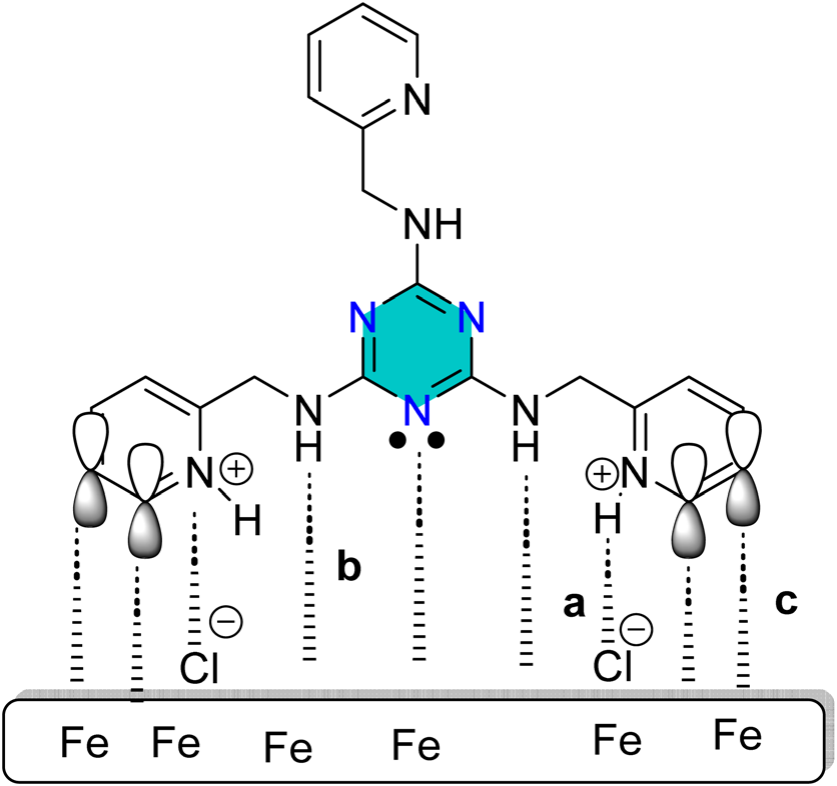
A proposed Schematic illustrating the adsorption mechanism of the 1,3,5-triazines derivative on steel surface in 1 M HCl: (a) physical adsorption; (b) and (c) chemical adsorption.

The physical adsorption of triazine derivatives onto the carbon steel surface mainly arises from electrostatic interactions between the protonated inhibitor molecules and the negatively charged steel surface as indicated in Fig. 21.^92,150^

Iron cations (Fe^2+^) are formed as Fe atoms on the steel surface undergo oxidation due to the attack of an acid HCl, or H_2_SO_4_. Chloride (Cl^−^) or sulfate anions (SO_4_^2−^) from the acid are attracted to positively charged sites containing Fe^2+^, resulting in the steel surface acquiring a net negative charge. Protonation of triazine derivative produces positively charged triazonium species. These protonated molecules are then strongly adsorbed onto the negatively charged steel surface through electrostatic forces.

In addition to physical adsorption (Fig. 21), chemical adsorption contributes significantly to the inhibition process. This occurs through the formation of σ-coordinate bonds between electron-rich functional groups in the triazine derivatives and the vacant d-orbitals of surface Fe or Fe^2+^ ions. Also, sites such as the imine group (>C=N) within triazine and side heterocyclic chain and ring such as pyridine or pyrazole, and the aromatic >C=C< bonds of phenyl groups, can donate π-electrons to Fe or Fe^2+^ (Fig. 21). The substituent effects enhance this interaction: methyl groups act as electron donors (*via* inductive effects), thereby increasing electron density of functional sites. Similarly, >N= and N< moieties can provide lone-pair electrons, in addition, donor groups (–Br and –OMe), which increase their ability to coordinate with Fe. Furthermore, π-back-donation interactions may reinforce adsorption. Electron density from the Fe (d) orbital can be transferred into the antibonding π* orbitals of the aromatic rings in triazine, phenylamine, and diazole structures.^101^

The calculated standard Gibbs free energies (Δ*G*° = −24 to −36 kJ mol^−1^) lie in the transitional region commonly interpreted as a combination of physical and chemical adsorption; therefore, the adsorption process is best described as mixed-type (comprehensive) adsorption. This interpretation is consistent with previous thermodynamic analyses that associate Δ*G*° values of this magnitude with contributions from both weak van-der-Waals/electrostatic forces and stronger specific interactions. Physical adsorption occurs due to interaction between protonated triazines and negatively charged iron surface which attracted Cl^−^ and SO_4_^2−^ from acids. While chemical adsorption occurs due to the nitrogen heteroatoms (lone pairs electrons) and the π system of the triazine ring (p-electrons) can contribute as electrons donor to the acceptor vacant d-orbital of Fe and Fe^2+^ on the surface of steel. The π system of the triazine ring can also participate in π–π stacking with aromatic adsorbates and can engage in partial charge-transfer interactions that strengthen adsorption beyond purely physical forces. Finally, electron density from the Fe (d) orbital can be transferred into the antibonding π* orbitals of the aromatic rings such as triazine or phenyl rings through p-back donation. The coexistence of these weak (dispersion, electrostatic) and stronger (donor–acceptor, π-backdonation) contributions rationalizes Δ*G*° values in the −24 to −36 kJ mol^−1^ range and supports the mixed-type assignment.

Experimental techniques are valuable for understanding the adsorption and inhibition behavior of these compounds. An increase in the inhibitor concentration, observed through weight loss tests, provides evidence for the corrosion inhibitor's adsorption.^151^ Electrochemical impedance spectroscopy (EIS) measurements show an increase in charge transfer resistance with increasing inhibitor concentrations.^152^ This indicates that the inhibitor displaces pre-adsorbed water molecules and other ions, leading to a decrease in the local dielectric constant and an increase in the thickness of the double layer, which enhances resistance at the metal-solution interface and lowers double-layer capacitance.^151^ Potentiodynamic polarization (PDP) studies, showing a reduction in corrosion current densities, also support inhibitor adsorption and can reveal the type of inhibition (cathodic, anodic, or mixed-type). Adsorption isotherms and standard free energy of adsorption values can further indicate the strength and spontaneity of the metal-inhibitor interaction.^151^ In summary, triazine-based corrosion inhibitors function by adsorbing onto metal surfaces through both physical (electrostatic) and chemical interactions,^153^ forming a protective film. Their molecular structure, including the arrangement of nitrogen atoms and the presence of various substituent groups, plays a crucial role in their adsorption behavior and overall inhibition performance, which can be elucidated through a combination of experimental and computational methods.

### 1.5. Role of substituent groups in triazine corrosion inhibitors

Substituent groups play a decisive role in enhancing the adsorption and corrosion inhibition efficiency of triazine-based inhibitors (Table 5). The introduction of various functional moieties, such as alkyl chains, –NH– groups, and additional heterocyclic units (*e.g.*, pyridine rings), can significantly improve the inhibitor's ability to adhere to the metal.^92,101^

**Table 5:** Summary for functional groups and their effects on corrosion inhibition

Functional group	Effect on corrosion inhibition	Mechanism/notes
Amines, amides	Increase adsorption, electron donation	Coordination, hydrogen bonding^155,156^
Hydroxyl (–OH)	Enhances hydrogen bonding, surface binding	Stronger metal-inhibitor interaction^156^
Carboxyl (–COOH)	Provides active sites, but excess may reduce efficiency	Adsorption, possible steric effects^83,106^
Nitrile, indole, methoxy	Promote electron density, surface interaction	Enhanced adsorption^100,101,157^
Alkyl chains	Longer chains increase surface coverage	Improved inhibition efficiency^83,106^
Phenyl rings, π-bonds	Facilitate π–π interactions, adsorption	Stronger surface binding^84,85,101,102^

The presence of functional groups and varying alkyl side chains length has a significant influence both adsorption and overall corrosion inhibition performance.^83,88,154^ For instance, extending the hydrocarbon chain length attached to triazine derivatives has been observed to promote the formation of a thicker and more compact protective layer on the metal surface, leading to superior inhibition efficiency.^64,83^

Substituent groups, whether electron-donating or electron-withdrawing, can significantly improve the adsorption of triazine molecules on the target metallic substrate.^91,101^ Studies have shown that electron-donating substituents generally lead to better inhibition performance compared to electron-withdrawing ones.^65,101–103^ The presence of these groups also influences the reactivity sites within the inhibitor molecules, which are critical for effective inhibition.

The presence of heteroatoms (like nitrogen, sulfur, and oxygen) and aromatic rings, along with π-bond conjugation, also plays a considerable role in the inhibitor's efficacy and adsorption behavior.^37–40,65,66,83,88,152–154^

Substituent groups affect both the physical (electrostatic) and chemical adsorption mechanisms. For example, the symmetric distribution of the three nitrogen atoms within the triazine ring promotes a nearly planar adsorption geometry on the metal surface, thereby enhancing surface coverage. The addition of specific substituent groups further enhances this coverage and the strength of the metal-inhibitor interaction.^83^ This can also influence whether the inhibitor absorbs in a neutral or protonated form.^83^

### 1.6. Conclusion, challenges and future perspectives

Triazine derivatives have gained considerable recognition as effective heterocyclic corrosion inhibitors. Numerous reports in the literature documents the successful application of triazine-based compounds in mitigating the aqueous corrosion of various metals and alloys under both acidic and neutral conditions. The remarkable inhibition performance is largely attributed to the structural characteristics of the triazine core, particularly the symmetrical distribution of three nitrogen atoms within the six-membered ring. Additionally, many studies indicate that the presence of substituents significantly enhances the corrosion inhibition behavior.

This geometric configuration allows for exceptional adsorption capabilities of the resulting organic molecules onto metallic substrates. Triazine-derived inhibitors are capable of undergoing both physisorption and chemisorption on metal surfaces.

In addition, this review explores potential application domains for triazine-derived corrosion inhibitors. These include alkaline environments, neutral environments with and without salinity, such as those simulating cooling water systems under heat exchange conditions, and saline environments saturated with carbon dioxide to replicate sweet corrosion scenarios. In mineral acids, particularly 15% HCl and 15–28% H_2_SO_4_, further exploration is necessary to simulate acidizing environments. Additionally, the application of triazine-based corrosion inhibitors in organic acid media and concrete environments warrants investigation.

Moreover, the effect of synergistic agents on triazines should be explored, alongside the potential of using fresh and expired triazine-based drug molecules for inhibition purposes. A significant advantage of triazines is that their synthesis can often be achieved under ambient conditions using low-toxicity reagents. Several reports detail multicomponent reactions and modern synthesis techniques, including microwave and ultrasound-assisted methods.

To comply with severe environmental guidelines for corrosion inhibitors, a full analysis of the green metrics associated with the synthesis methodologies of triazine-based corrosion inhibitors is essential. Furthermore, toxicity tests on animal models are necessary to get reliable toxicity data.

#### 1.6.1. Challenges and future perspectives for triazine corrosion inhibitors

Despite their effectiveness, existing organic corrosion inhibitors, including some triazine derivatives, face several challenges:

(1) Synthesis complexity: the synthesis of many current corrosion inhibitors, such as imidazolines, acetylenic alcohols, amides, amines, and quaternary-ammonium salts, is often a cumbersome procedure involving complex synthetic steps, which are time-consuming and costly. Moreover, the separation and purification of these inhibitors can be difficult.^2^

(2) Environmental concerns: the release of these inhibitors into the soil and aquatic environments poses a significant environmental concern.^158^ There is a need to meet strict environmental guidelines, requiring thorough analysis of the “green metrics” of synthetic protocols and reliable toxicity data from animal model tests.^159^

(3) Lack of data in specific media: while triazines have been extensively studied in acidic media, there is a notable lack of reports concerning their application on mild steel and carbon steel in neutral media.

#### 1.6.2. Future perspectives and research directions

Future research on triazine-derived corrosion inhibitors should focus on expanding their application and addressing environmental concerns:

(1) Broader environmental applications: prospective application areas include alkaline environments, neutral environments with and without salinity representative of cooling water and heat-exchange systems as well as saline solutions saturated with carbon dioxide to simulate sweet corrosion conditions.^160^

(2) Specific acidic environments: in mineral acids, further exploration is needed in 15% HCl and 15–28% H_2_SO_4_ to better simulate acidizing environments.

(3) Novel media and synergistic effects: the potential application of triazine-based corrosion inhibitors in organic acid media and concrete environments requires exploration. Furthermore, the influence of synergistic agents in combination with triazines needs to be investigated, and the potential use of fresh and expired triazine-based drug molecules for inhibition should be explored.

(4) Sustainable synthesis and safety: a major advantage of triazines is that their synthesis can often be achieved under ambient conditions using low-toxicity reagents, with many reports on multicomponent reactions and modern techniques like microwave and ultrasound-based synthesis. However, to comply with environmental guidelines, a thorough analysis of the green metrics of their synthesis protocols and toxicity tests on animal models are crucial to obtaining reliable toxicity data.

In summary, while triazine derivatives show great promise as corrosion inhibitors, future efforts must address the challenges of complex synthesis and environmental impact, while expanding their tested applications across a wider range of corrosive environments and exploring synergistic effects.

## Author contributions

Ayman El-Faham, Hassan Hammud, and Assem Barakat: conceptualization, investigation, writing – original draft, writing – review & editing. Ihab Shawish and Hessa Al-Rasheed: writing – original draft, writing – review & editing. Ihab Shawish and Hassan Hammud: funding acquisition. Ayman El-Faham, Hassan Hammud, and Assem Barakat: validation, supervision. All authors read and approved the submitted version.

## Conflicts of interest

The authors declare no conflict of interest.

## Data Availability

No new data generated as this is a review.
